# Supplementary material for: Pan-cancer landscape of homologous recombination deficiency
Source: Nat Commun. 2020 Nov 4;11:5584. doi: 10.1038/s41467-020-19406-4 (PMC7643118; doi:10.1038/s41467-020-19406-4)
Supplement: Supplementary file 1 — Supplementary Information [file 41467_2020_19406_MOESM1_ESM.pdf]

Supplementary tables

Cancer type	HRD patients with *BRCA1* promoter methylation (data from Davies et al. 2017)	CHORD-HRD patients without biallelic loss of HRD associated genes
Ovarian	22% (10/46)	47% (36/76)
Breast	16% (14/86)	49% (50/103)

Supplementary table 1: Comparing prevalence of *BRCA1* promoter methylation in HRDetect-HRD patients with CHORD-HRD patients without biallelic loss of *BRCA1/2*, *RAD51C* or *PALB2*

Supplementary figures

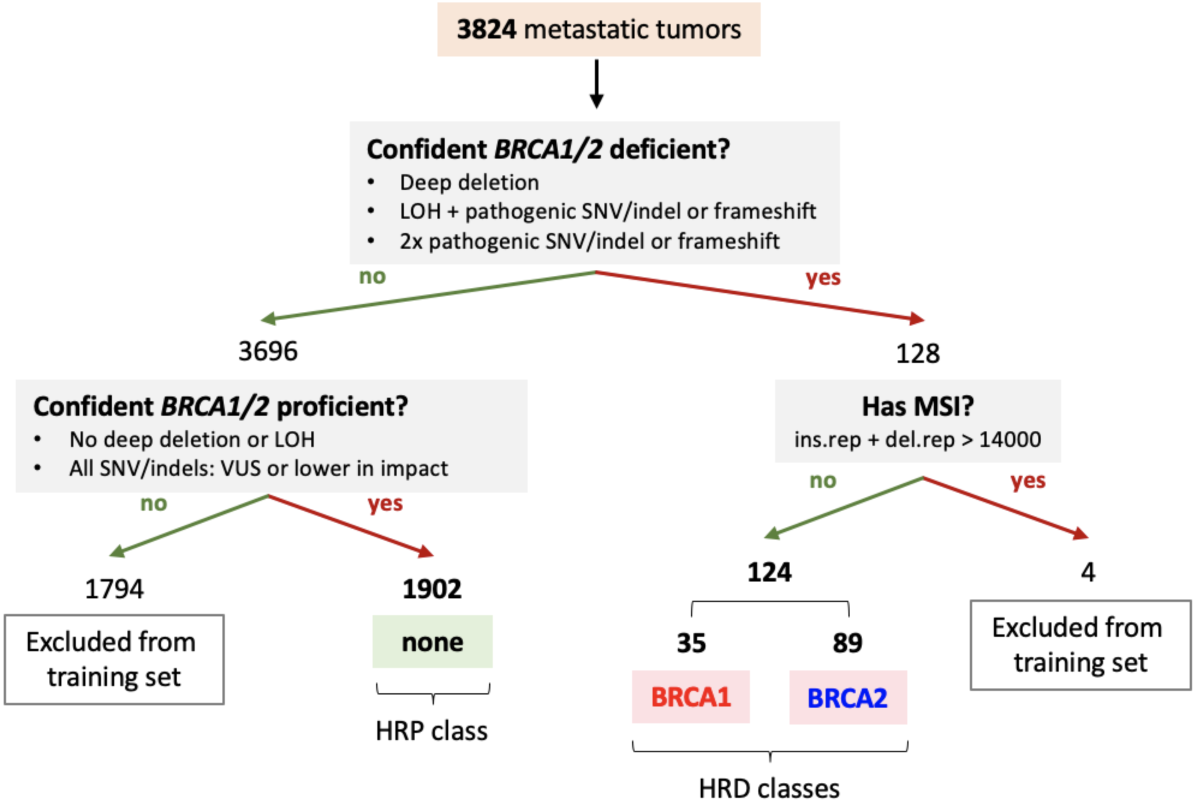

Supplementary figure 1: Sample selection for training CHORD. Only samples where biallelic inactivation of *BRCA1/2* could confidently be identified (and did not have MSI), or where no disruption of *BRCA1/2* was apparent were recruited into the training set. A total of 2,026 samples were eventually selected, consisting of 35 *BRCA1* and 89 *BRCA2* deficient which were both considered HRD during training, and 1,902 *BRCA1/2* proficient (*'none'*) which were considered HRP. LOH: loss-of-heterozygosity; VUS: variant of unknown significance

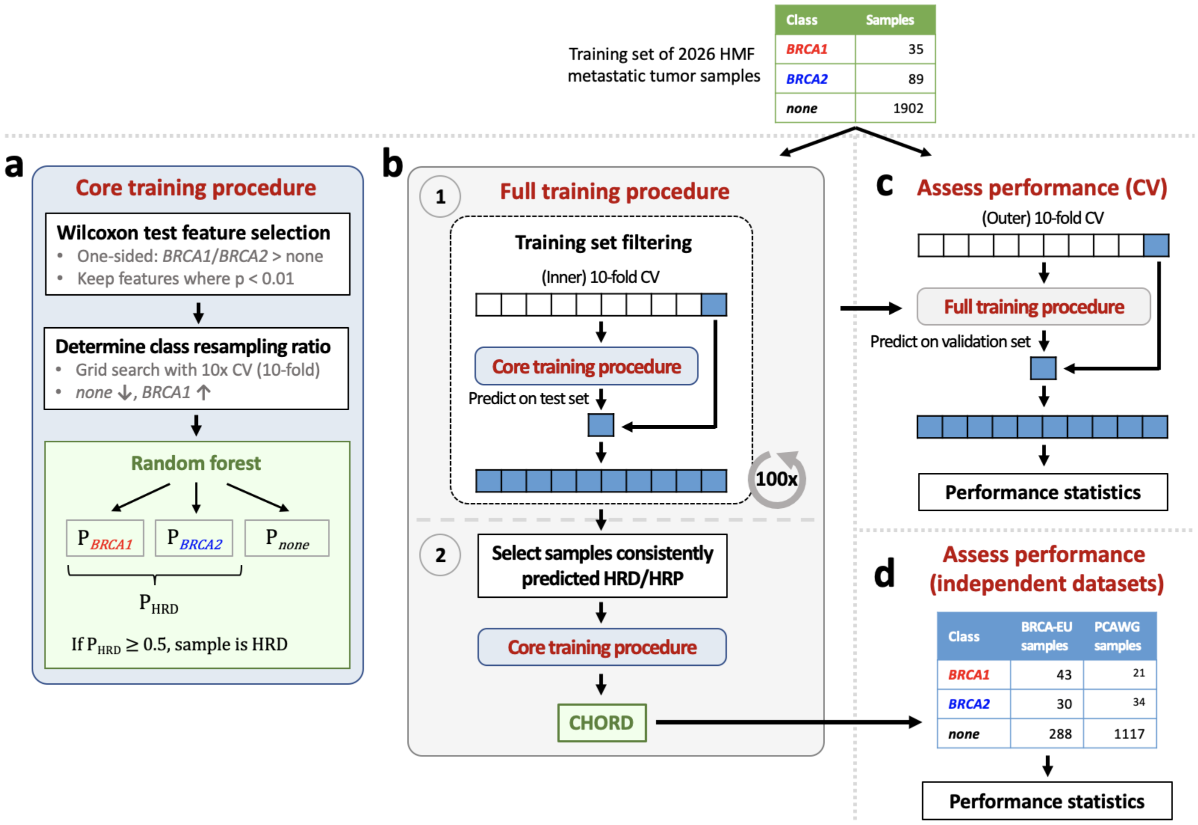

Supplementary figure 2: Workflow for training CHORD. *'BRCA1'*, *'BRCA2'* and *'none'* classes refer to *BRCA1* deficient, *BRCA2* deficient and *BRCA1/2* proficient sample groups, respectively. (**a**) The core training procedure on which the full training procedure is based on performs feature selection and class resampling. This returns a random forest that outputs the probability of a new sample being in one of the aforementioned 3 classes. The probability of HRD (P_{HRD}) is the sum of the probability of belonging to the *'BRCA1'* and *'BRCA2'* classes, where a sample is considered HRD if P_{HRD} is ≥ 0.5. (**b**) The full training procedure is split into 2 stages. The first stage serves to blacklist *'BRCA1'* or *'BRCA2'* class samples which are likely not HRD (e.g. due to reversal of biallelic inactivation via a secondary frameshift), or *'none'* samples which are likely not HRP (e.g. due to deficiencies in other HR genes). Here, the core training procedure is encapsulated by a 10-fold cross-validation (CV) loop to allow every sample to be excluded from the training set to subsequently calculate an unbiased P_{HRD}. This was repeated 100 times and the number of times each sample was HRD or HRP was calculated. *'BRCA1'* or *'BRCA2'* samples that were predicted HRD < 60 times were blacklisted. *'none'* samples that were predicted HRD > 40 times were blacklisted. In the second training stage, the core training procedure was performed on a training set without the blacklisted samples. This produced the final random forest model, CHORD. (**c**) The full training procedure was further encapsulated by a 10-fold CV to assess the performance of CHORD. (**d**) Performance was also assessed by applying CHORD on two independent datasets: BRCA-EU (371 primary breast tumors) and PCAWG (1176 primary tumors, pan-cancer).

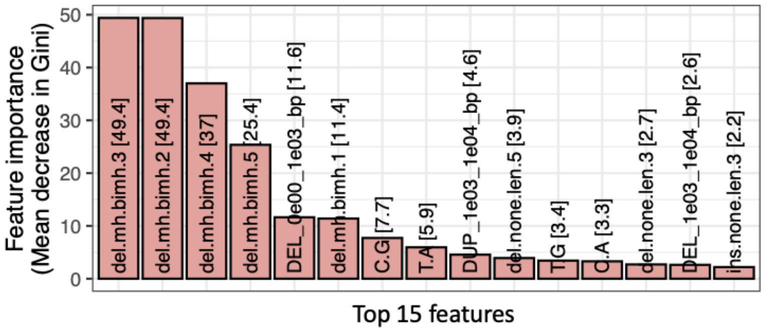

Supplementary figure 3: Deletions with ≥2bp flanking homology were most predictive of *BRCA1/2* deficiency. A random forest was applied to the training set and the importance of each feature was quantified using the mean decrease in Gini. The features used for training are relative counts of different mutation contexts which fall into one of three groups based on mutation type. (i) Single nucleotide variants (SNV): 6 possible base substitutions (C>A, C>G, C>T, T>A, T>C, T>G). (ii) Indels: indels with flanking microhomology stratified by homology length (del.mh, ins.mh), within repeat regions (del.rep, del.none), or not falling into either of these 2 categories (del.none, ins.none). (iii) Structural variants (SV): SVs stratified by type and length.

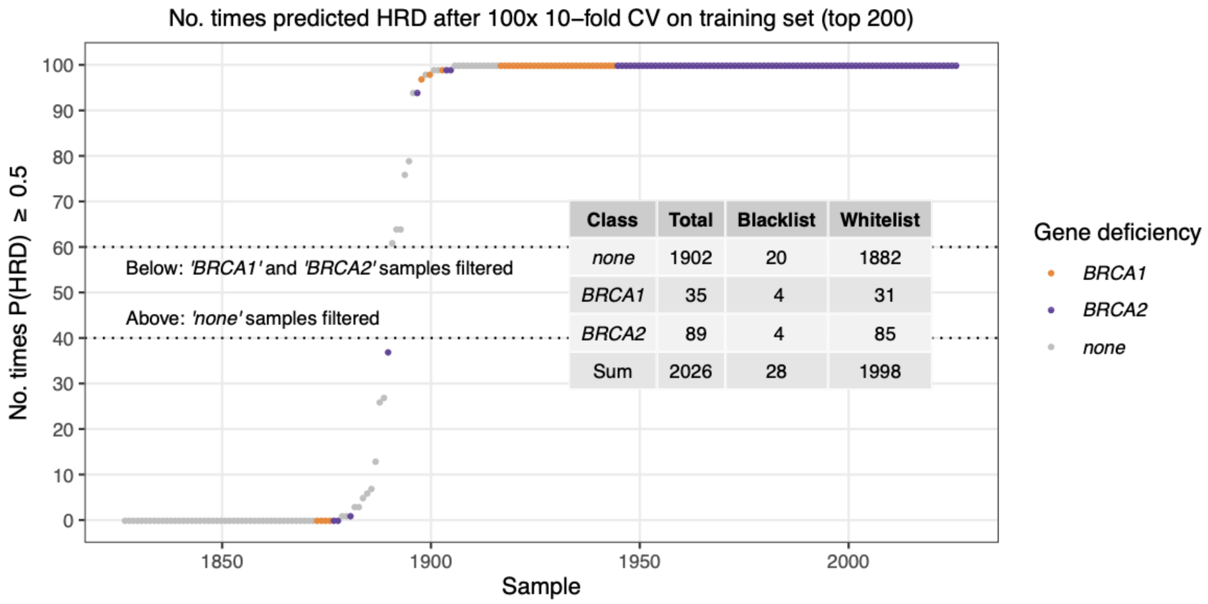

Class	Total	Blacklist	Whitelist
none	1902	20	1882
BRCA1	35	4	31
BRCA2	89	4	85
Sum	2026	28	1998

Supplementary figure 4: **Results of the 100x repeated 10-fold CV procedure for filtering CHORD training samples.** This was performed to blacklist *'BRCA1'* or *'BRCA2'* class samples that were consistently predicted HRP (no. times HRD < 60), or *'none'* class samples that were consistently predicted HRD (no. times HRD > 40). Samples with a probability of HRD ≥ 0.5 were considered HRD. The overlaid table summarizes the number of samples per class before and after removing the blacklisted samples from the training set. Note that only the top 200 samples are shown here as the remaining samples belonged to the *'none'* class and were always predicted HRP.

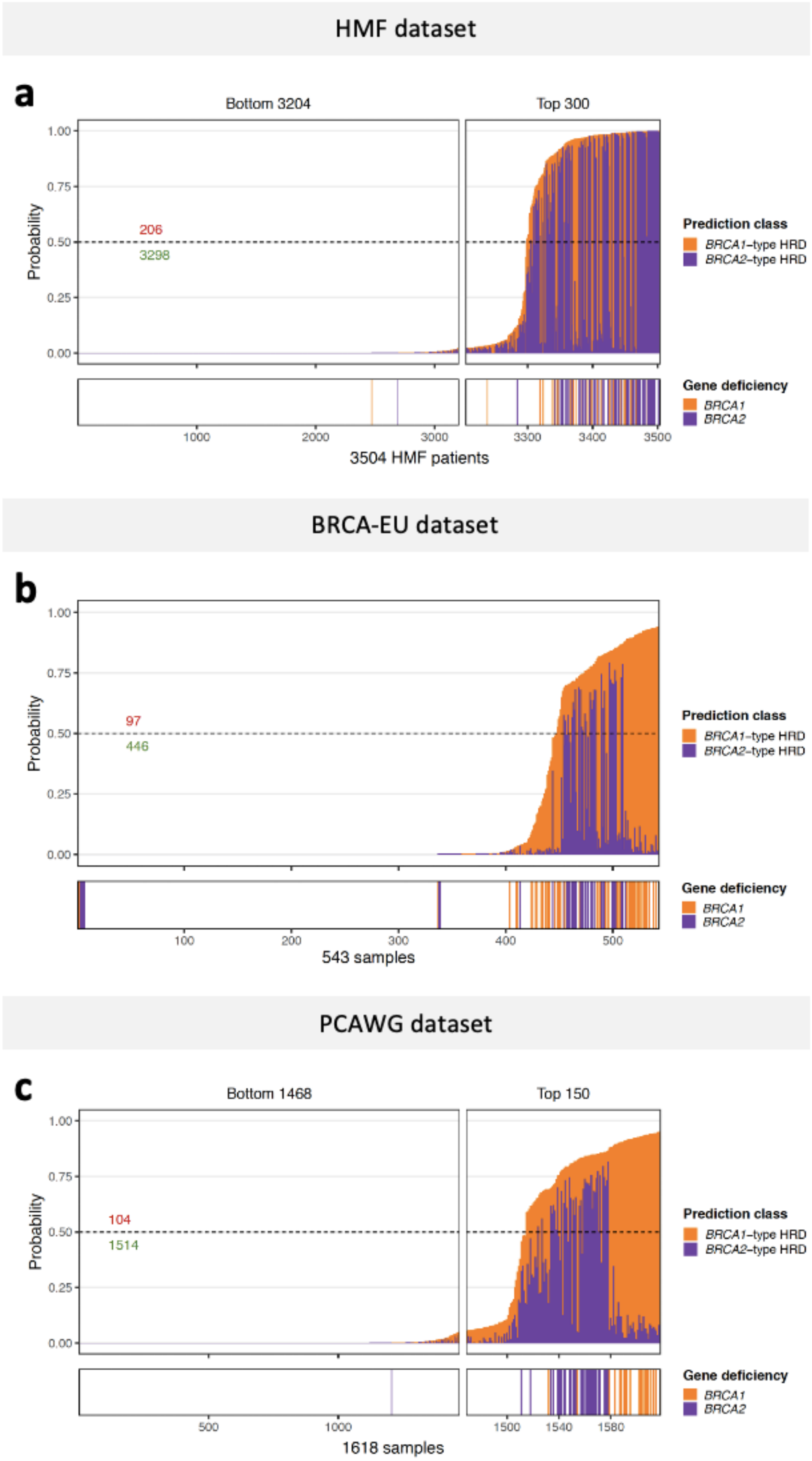

Supplementary figure 5: CHORD predictions on all HMF, BRCA-EU and PCAWG patients. Predictions on the HMF, BRCA-EU and PCAWG datasets are shown in **a**, **b** and **c**, respectively. The probability of HRD for each sample (total bar height) with each bar being divided into segments indicating the probability of *BRCA1*- (orange) and *BRCA2*-type HRD (purple). Stripes below the bar plot indicate biallelic loss of *BRCA1* or *BRCA2*. Only samples that passed CHORD's QC criteria are shown (MSI negative, ≥50 indels, and ≥30 SVs if a sample was predicted HRD)

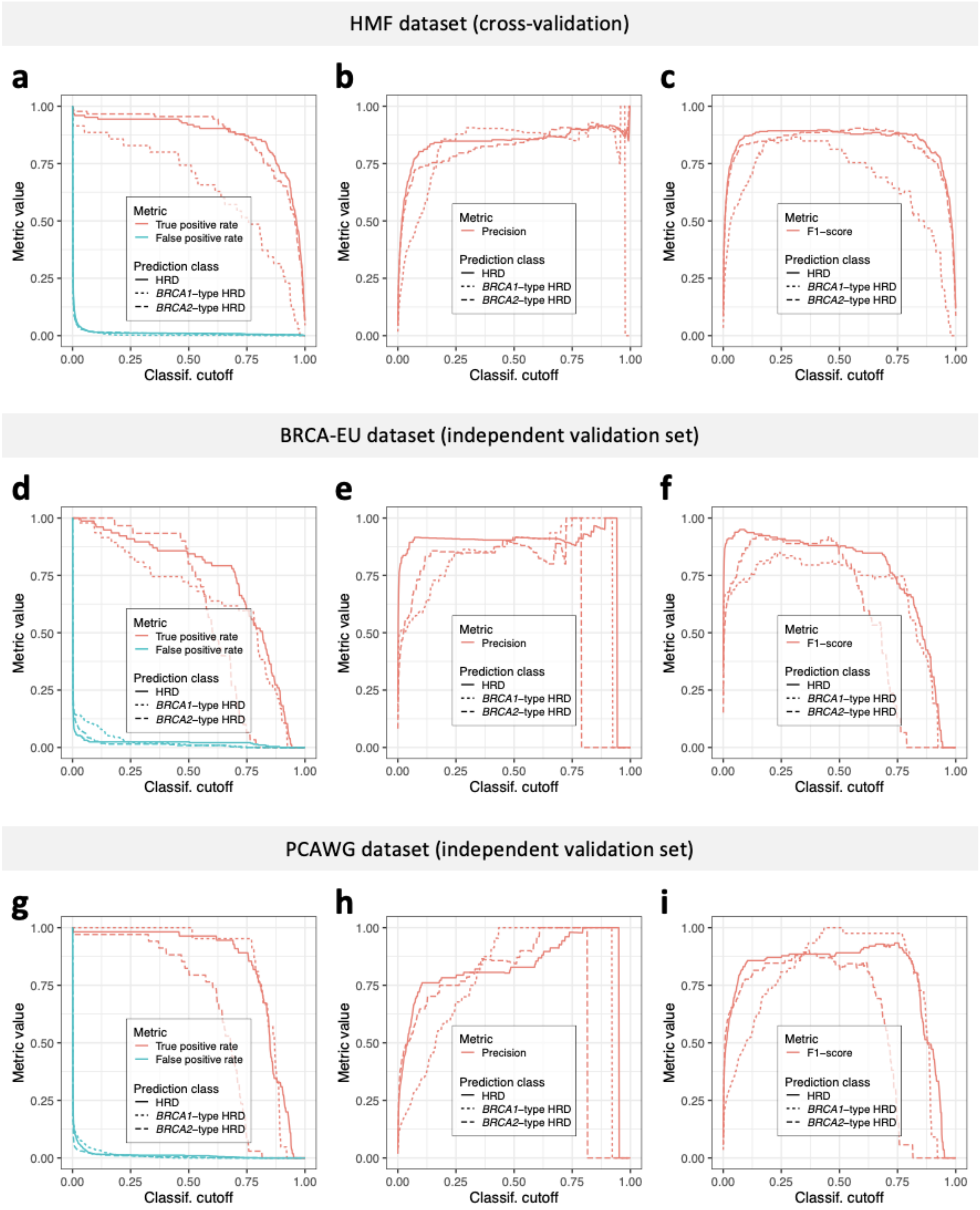

Supplementary figure 6: Additional performance metrics for CHORD. Performance was determined by 10-fold cross-validation (CV) on the HMF training data (**a-c**) or prediction on two independent datasets, BRCA-EU (primary breast cancer dataset; **d-f**) and PCAWG (primary pan-cancer dataset; **g-i**). Data from the BRCA-EU and PCAWG datasets are from samples that passed CHORD's QC criteria (i.e. MSI absent, ≥50 indels, ≥30 SVs if a sample was predicted HRD).

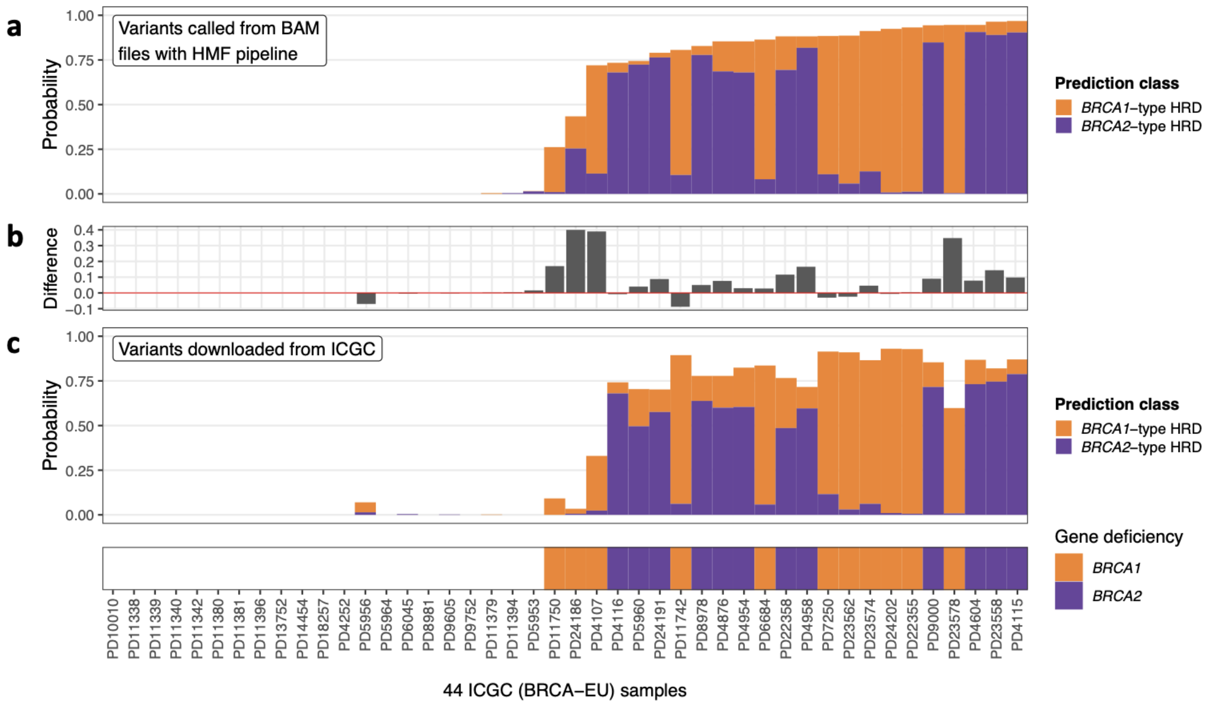

Supplementary figure 7: Variant calling pipeline differences affects CHORD performance. (**a**) Using variants called with the native pipeline of the HMF dataset (HMF pipeline) for HRD prediction with CHORD resulted in overall higher HRD probabilities in *BRCA1/2* deficient tumors when compared to (**c**) using variants downloaded from ICGC. The differences in HRD probabilities are quantified in (**b**). All samples shown here passed CHORD's QC criteria (i.e. MSI absent, ≥50 indels, ≥30 SVs if a sample was predicted HRD)

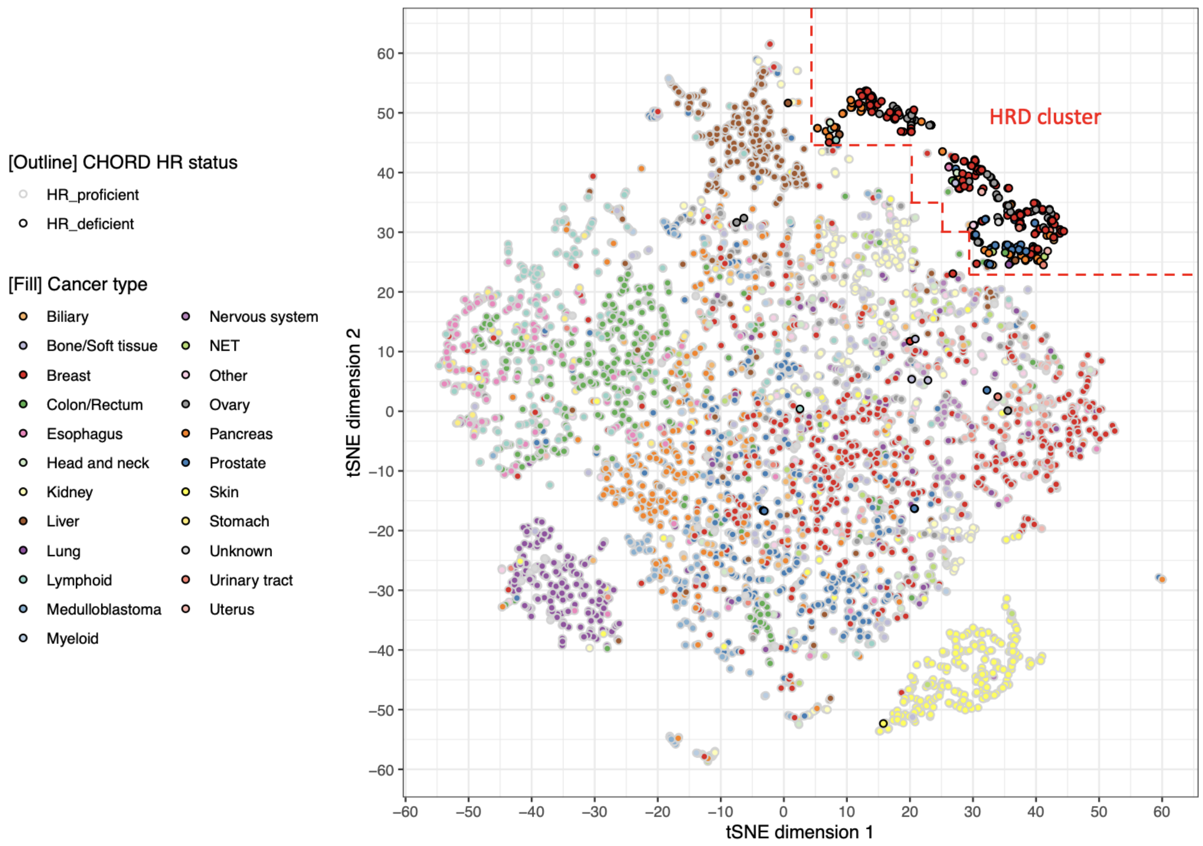

Supplementary figure 8: Clustering of samples (HMF, n=2026; PCAWG, n=1854) by t-distributed stochastic neighbor embedding (t-SNE) on the features used as input for CHORD. The dashed red lines are a manual annotation of the HRD cluster. All samples shown here passed CHORD's QC criteria (i.e. MSI absent, ≥50 indels, ≥30 SVs if a sample was predicted HRD).

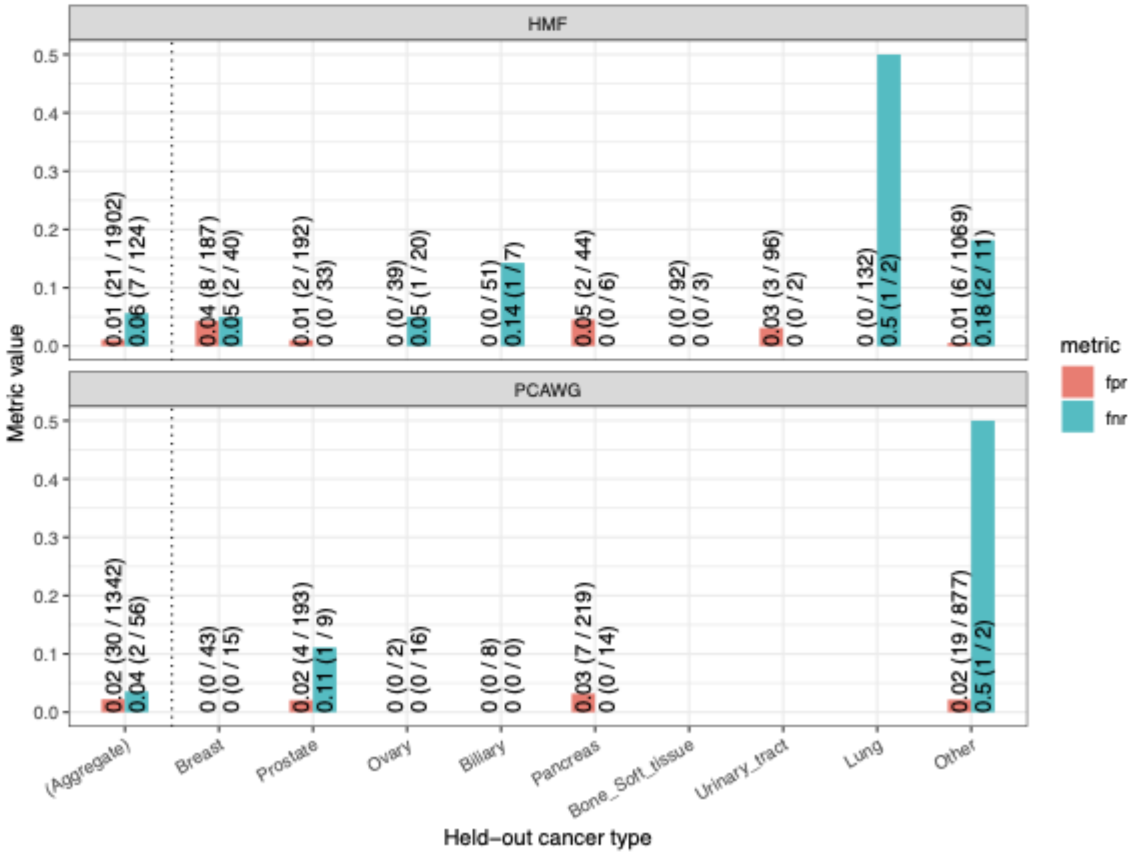

Supplementary figure 9: Performance of random forests trained when HMF samples belonging to each cancer type were held out. Training was performed in the same manner as for CHORD. These models were then applied to the held out HMF samples as well as the PCAWG samples to calculate performance metrics (at a classification threshold of 0.5). Only samples where *BRCA1/2* deficiency status could be confidently determined were included in this analysis (HMF, n=2026; PCAWG, n=1854). False positive rate (FPR) = number of *BRCA1* or *BRCA2* proficient samples misclassified as HR proficient / total number of *BRCA1* or *BRCA2* proficient samples. False negative rate (FNR) = number of *BRCA1* or *BRCA2* deficient samples misclassified as HR deficient / total number of *BRCA1* or *BRCA2* deficient samples. 'Aggregate' refers to the aggregated FPR and FNR across all cancer types. 'Other' refers to cancer types with <2 *BRCA1/2* deficient samples.

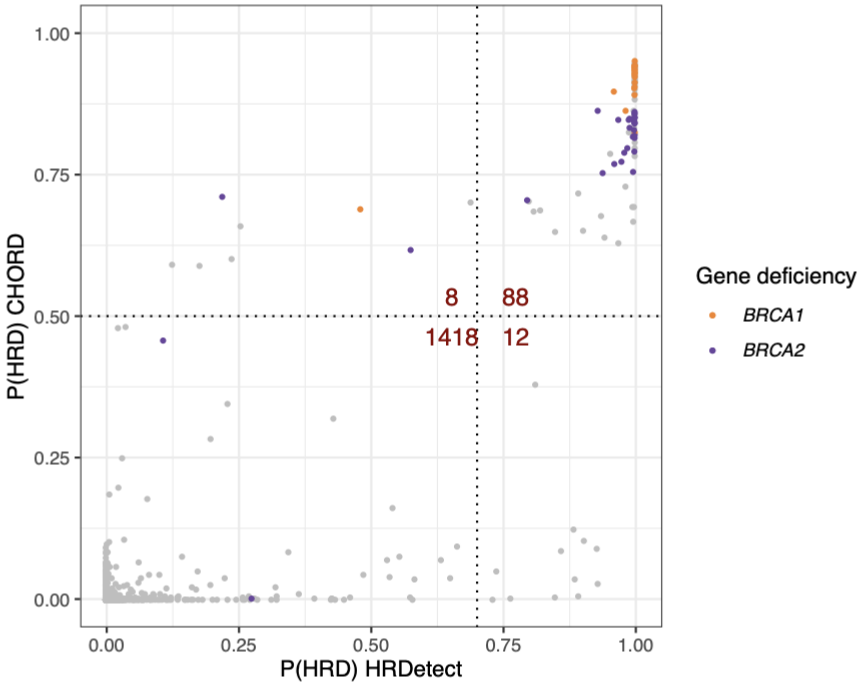

Supplementary figure 10: CHORD vs HRDetect predictions on the PCAWG dataset. The 1526 samples shown here had predictions from HRDetect and passed CHORD's QC criteria for predicting HRD (i.e. MSI absent, ≥50 indels, ≥30 SVs if a sample was predicted HRD). The dotted lines indicate the classification thresholds for the two models (CHORD: 0.5, HRDetect: 0.7). P(HRD): probability of HRD

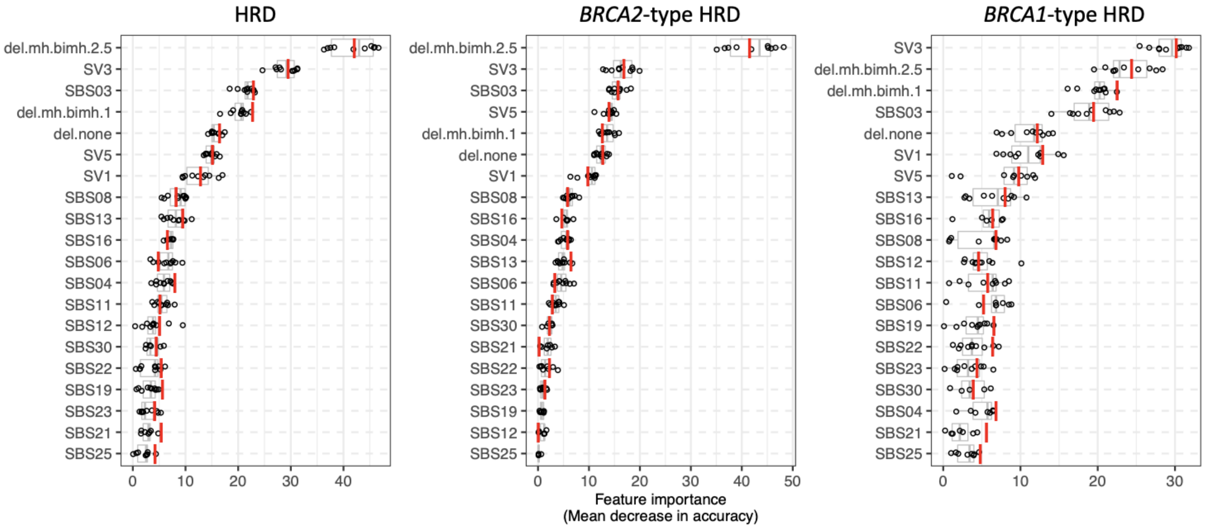

Supplementary figure 11: The features used by CHORD-signature to predict HRD as well as *BRCA1*-type HRD and *BRCA2*-type HRD. Importance is indicated by mean decrease in accuracy. SV# refers to the 6 SV mutational signatures and SBS# refers to the 30 single base substitution signatures as used by HRDetect. Indels are stratified into those with flanking microhomology further stratified by homology length (del.mh, ins.mh), within repeat regions (del.rep, del.none), or not falling into either of these 2 categories (del.none, ins.none). Boxplot and dots (n=10) show the feature importance over 10-folds of nested CV on the training set, with the red line showing the feature importance in the final CHORD model. Boxes show the interquartile range (IQR) and whiskers show the largest/smallest values within 1.5 times the IQR.

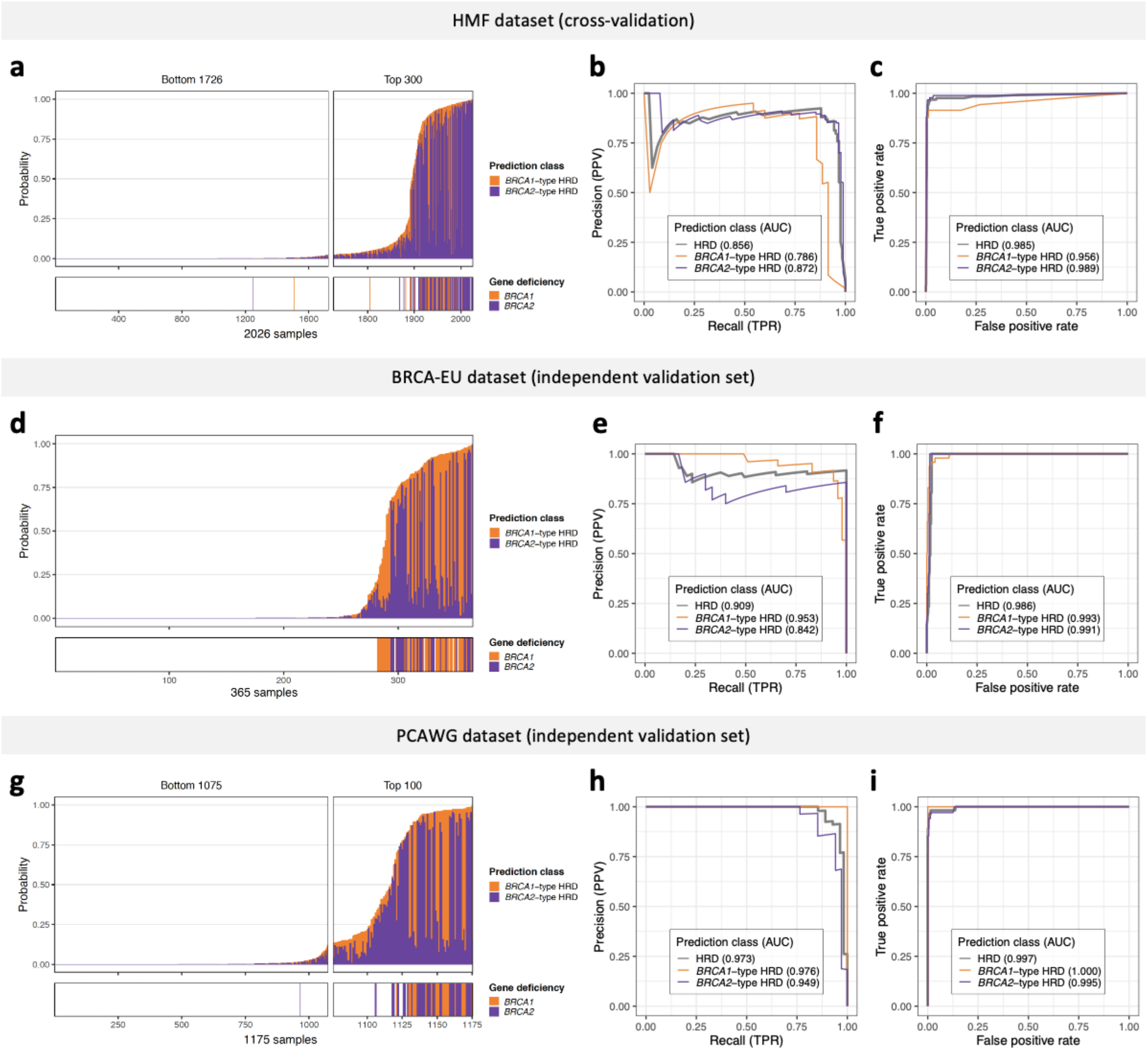

Supplementary figure 12: Performance of CHORD-signature. Performance was determined by 10-fold cross-validation (CV) on the HMF training data or prediction on two independent datasets: BRCA-EU (primary breast cancer dataset) and PCAWG (primary pan-cancer dataset). BRCA-EU and PCAWG samples shown here all passed CHORD's QC criteria (i.e. MSI absent, ≥50 indels, ≥30 SVs if a sample was predicted HRD) (**a, d, g**) The probability of HRD for each sample (total bar height) with each bar being divided into segments indicating the probability of *BRCA1*- (orange) and *BRCA2*-type HRD (purple). Stripes below the bar plot indicate biallelic loss of *BRCA1* or *BRCA2*. In (**a**), probabilities have been aggregated from the 10 CV folds. (**b, e, h**) Receiver operating characteristic (ROC) and (**c, f, i**) precision-recall curves (PR) and respective area under the curve (AUC) values showing the performance of CHORD when predicting HRD as a whole (grey), *BRCA1*-type HRD (orange), or *BRCA2*-type HRD (purple).

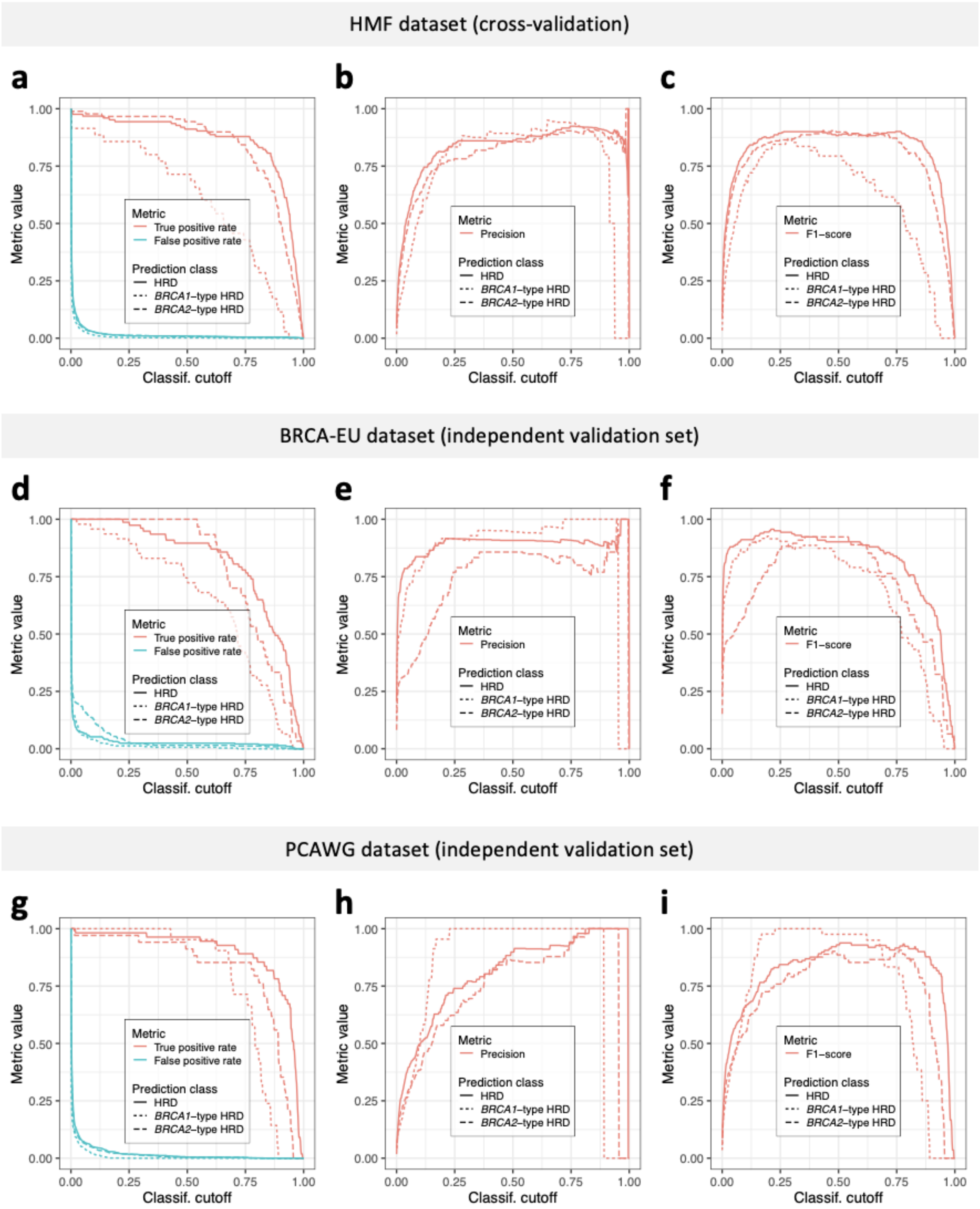

Supplementary figure 13: Additional performance metrics for CHORD-signature. Performance was determined by 10-fold cross-validation (CV) on the HMF training data (**a-c**) or prediction on two independent datasets, BRCA-EU (primary breast cancer dataset; **d-f**) and PCAWG (primary pan-cancer dataset; **g-i**). Data from the BRCA-EU and PCAWG datasets are from samples that passed CHORD's QC criteria (i.e. MSI absent, ≥50 indels, ≥30 SVs if a sample was predicted HRD).

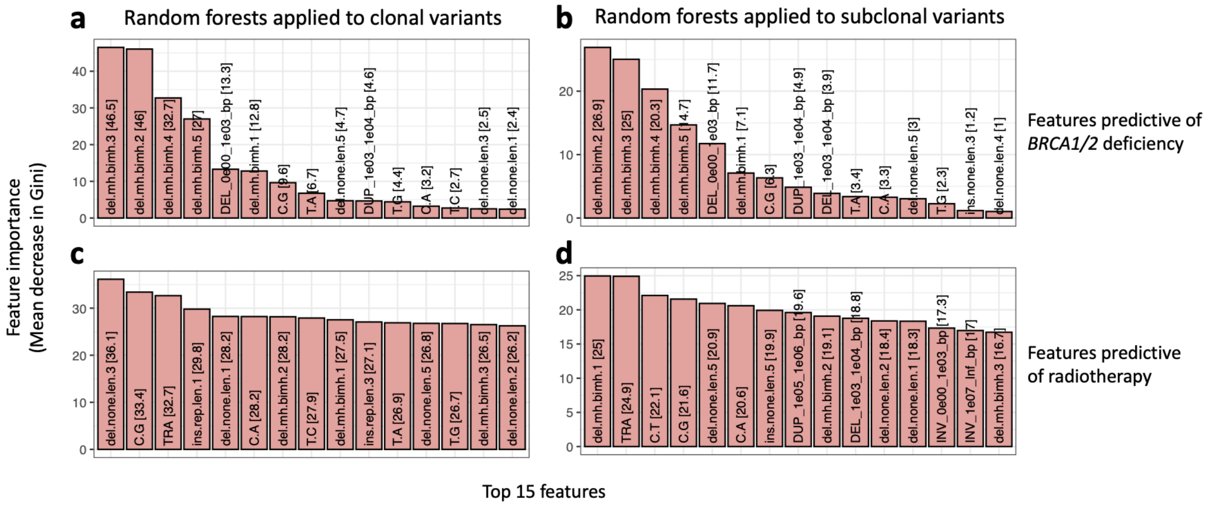

Supplementary figure 14: Random forests were used to identify the features predictive of *BRCA1/2* deficiency (a,b) compared to those predictive of radiotherapy (c,d) when using clonal versus subclonal variants as input. Feature importance is indicated by mean decrease in Gini, with only the top 15 important features being shown. The features used for training are relative counts of different mutation contexts which fall into one of three groups based on mutation type. (i) Single nucleotide variants (SNV): 6 possible base substitutions (C>A, C>G, C>T, T>A, T>C, T>G). (ii) Indels: indels with flanking microhomology stratified by homology length (del.mh, ins.mh), within repeat regions (del.rep, del.none), or not falling into either of these 2 categories (del.none, ins.none). (iii) Structural variants (SV): SVs stratified by type and length. Deletions with 1bp of flanking homology (del.mh.bimh.1) was more associated with radiotherapy especially in the subclonal fraction, while deletions with ≥2bp flanking homology (del.mh.bimh.2.5) was more associated with *BRCA1/2* deficiency.

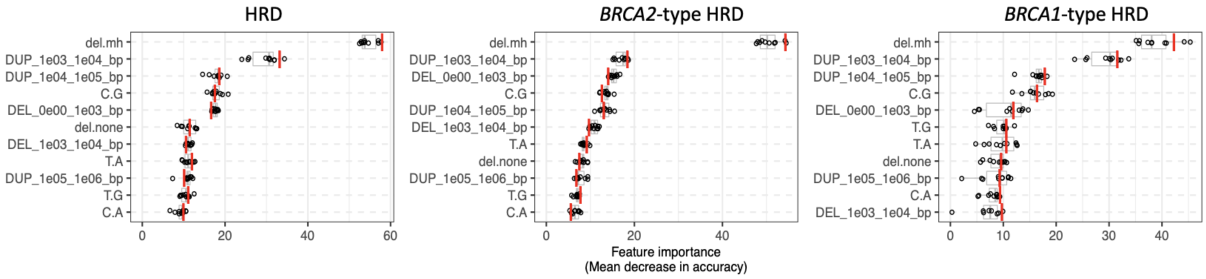

Supplementary figure 15: The features used by CHORD-del.mh.merged to predict HRD as well as *BRCA1*-type HRD and *BRCA2*-type HRD. Importance is indicated by mean decrease in accuracy. The features used for training are relative counts of different mutation contexts which fall into one of three groups based on mutation type. (i) Single nucleotide variants (SNV): 6 possible base substitutions (C>A, C>G, C>T, T>A, T>C, T>G). (ii) Indels: indels with flanking microhomology stratified by homology length (del.mh, ins.mh), within repeat regions (del.rep, del.none), or not falling into either of these 2 categories (del.none, ins.none). (iii) Structural variants (SV): SVs stratified by type and length. Deletions with flanking microhomology (del.mh) was the most important feature for predicting HRD as a whole, with 1-100kb structural duplications (DUP_1e03_1e04_bp, DUP_1e04_1e05_bp) differentiating *BRCA1*-type HRD from *BRCA2*-type HRD. Boxplot and dots (n=10) show the feature importance over 10-folds of nested CV on the training set, with the red line showing the feature importance in the final CHORD model. Boxes show the interquartile range (IQR) and whiskers show the largest/smallest values within 1.5 times the IQR.

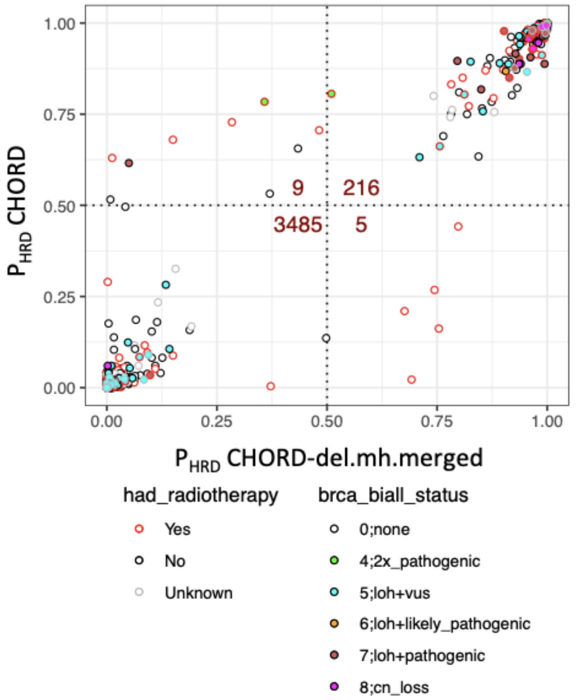

Supplementary figure 16: HRD predictions (on HMF samples) from CHORD and CHORD-del.mh.merged on all variants. Only samples passing CHORD's QC criteria were shown (MSI negative, ≥50 indels, and ≥30 SVs if a sample was predicted HRD). Dots with a red outline indicate samples which had radiotherapy prior to the tumor biopsy. Dot fill color indicates the biallelic status of *BRCA1* or *BRCA2*. LOH: loss-of-heterozygosity; VUS: variant of unknown significance; P_{HRD}: probability of HRD

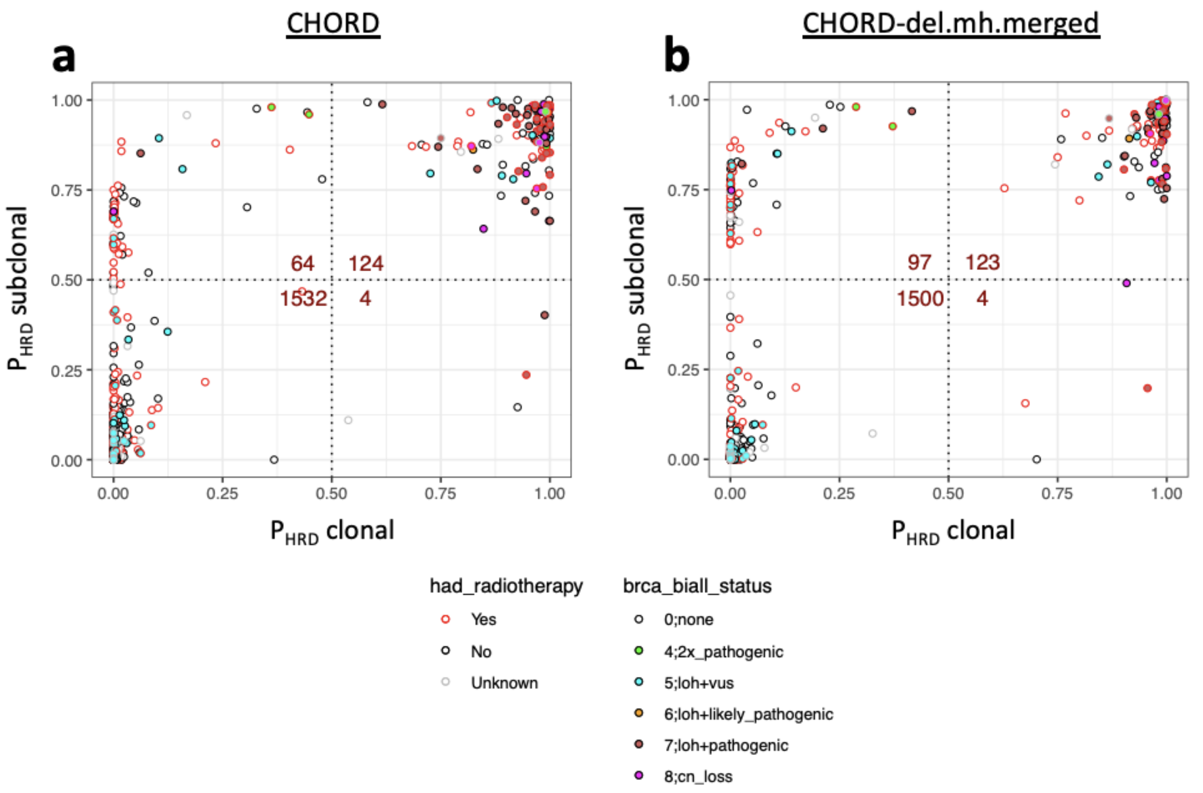

Supplementary figure 17: HRD predictions (on HMF samples) on subclonal versus clonal variants and from (a) CHORD and (b) CHORD-del.mh.merged. Only samples passing CHORD's QC criteria were shown (MSI negative, ≥50 indels in both clonal and subclonal fractions, and ≥30 SVs if a sample was predicted HRD). Dots with a red outline indicate samples which had radiotherapy prior to the tumor biopsy. Dot fill color indicates the biallelic status of *BRCA1* or *BRCA2*. LOH: loss-of-heterozygosity; VUS: variant of unknown significance; P_{HRD}: probability of HRD

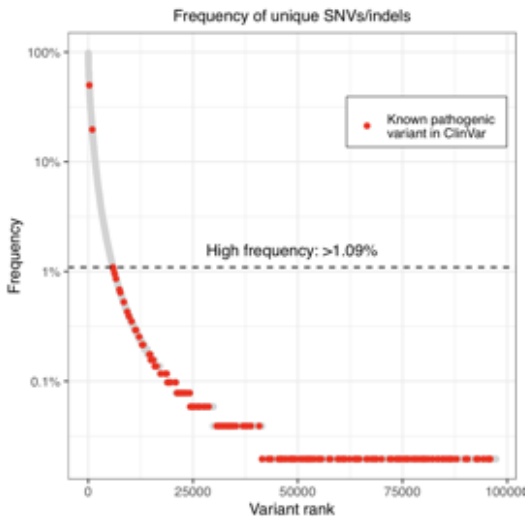

Supplementary figure 18: Frequency of unique germline SNVs/indels of 781 cancer related genes in patients of the HMF cohort. Germline variants with a frequency >1.09% were marked as benign prior to performing the pan-cancer analysis of HRD. Two known pathogenic variants above this frequency were also considered benign.

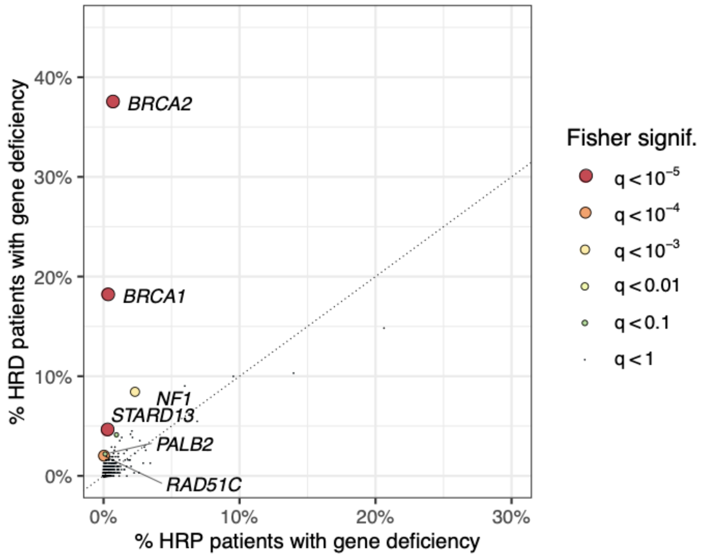

Supplementary figure 19: **A one-tailed Fisher's exact test identified enrichment of *BRCA1, BRCA2, RAD51C* and *PALB2* biallelic inactivation in CHORD-HRD vs. CHORD-HRP patients.** Each point represents a gene (from a list of 781 cancer and HR related genes) with its size/color corresponding to the statistical significance as determined by the Fisher's exact test, with axes indicating the percentage of patients (within either the CHORD-HRD or CHORD-HRP group) in which biallelic inactivation was detected. Multiple testing correction was performed using the Hochberg procedure. This figure is the same as **Figure 3b** except with *NF1* and *STARD13* included. A one-tailed Fisher's exact test determined *BRCA1* (q=9.4e-51), *BRCA2* (q=4.8e-101), *RAD51C* (q=5.6e-5), *PALB2* (q=0.02), *NF1* (q=3.5e-7) and *STARD13* (q=3.0e-8) to be significantly enriched (from a list of 781 cancer related genes) in CHORD-HRD vs. CHORD-HRP patient groups. Each point represents a gene with its size/color corresponding to the statistical significance as determined by the Fisher's exact test, with axes indicating the percentage of patients (within either the CHORD-HRD or CHORD-HRP group) in which biallelic inactivation was detected. Multiple testing correction was performed using the Hochberg procedure.

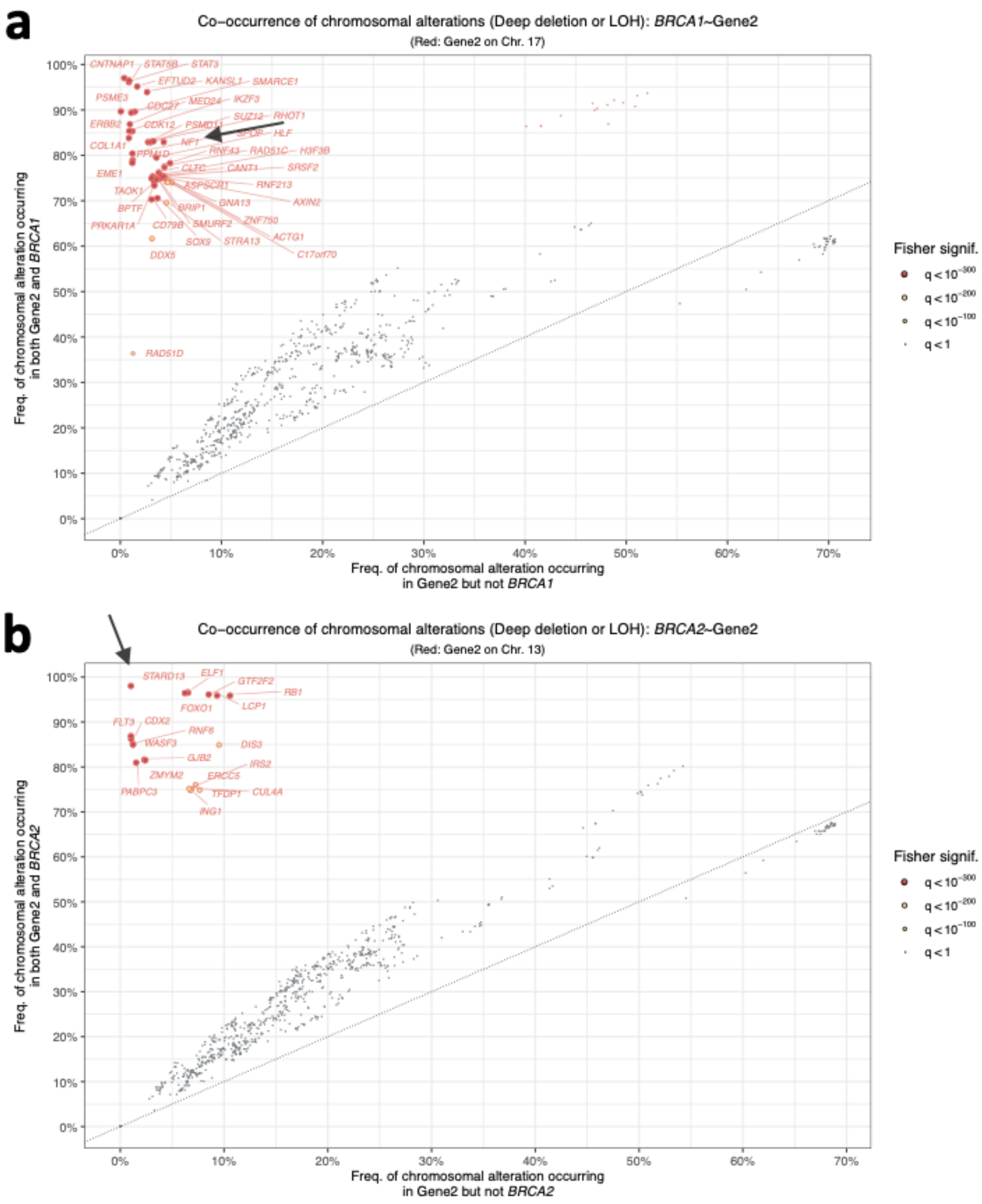

Supplementary figure 20: Chromosomal alterations (deep deletions or loss of heterozygosity (LOH)) affecting *BRCA1* and *BRCA2* also affects nearby genes including *NF1* and *STARD13* respectively. (**a**) Enrichment of *NF1* (q<1e-300) biallelic loss as shown in **Supplementary figure 19** is likely due to the gene being within proximity of *BRCA1* and not because the gene is associated with HRD, since *NF1* is not considered to be involved in HR in literature. The size/color of each point on the plot represents the significance of enrichment of CNAs occurring both in *BRCA1* and in each of the 781 genes (one vs. all comparison). This was determined by a one-tailed Fisher's exact test, where multiple testing correction was performed using the Hochberg procedure. Genes residing on the same chromosome as *BRCA1* were marked with a red outline and text. (**a**) Similarly, as with (**b**), a chromosomal alteration affecting *BRCA2* also affects the nearby gene *STARD13* (q<1e-300).

Enrichment of *STARD13* biallelic loss as shown in **Supplementary figure 19** is likely due to the gene being within proximity of *BRCA2* and not because the gene is associated with HRD.

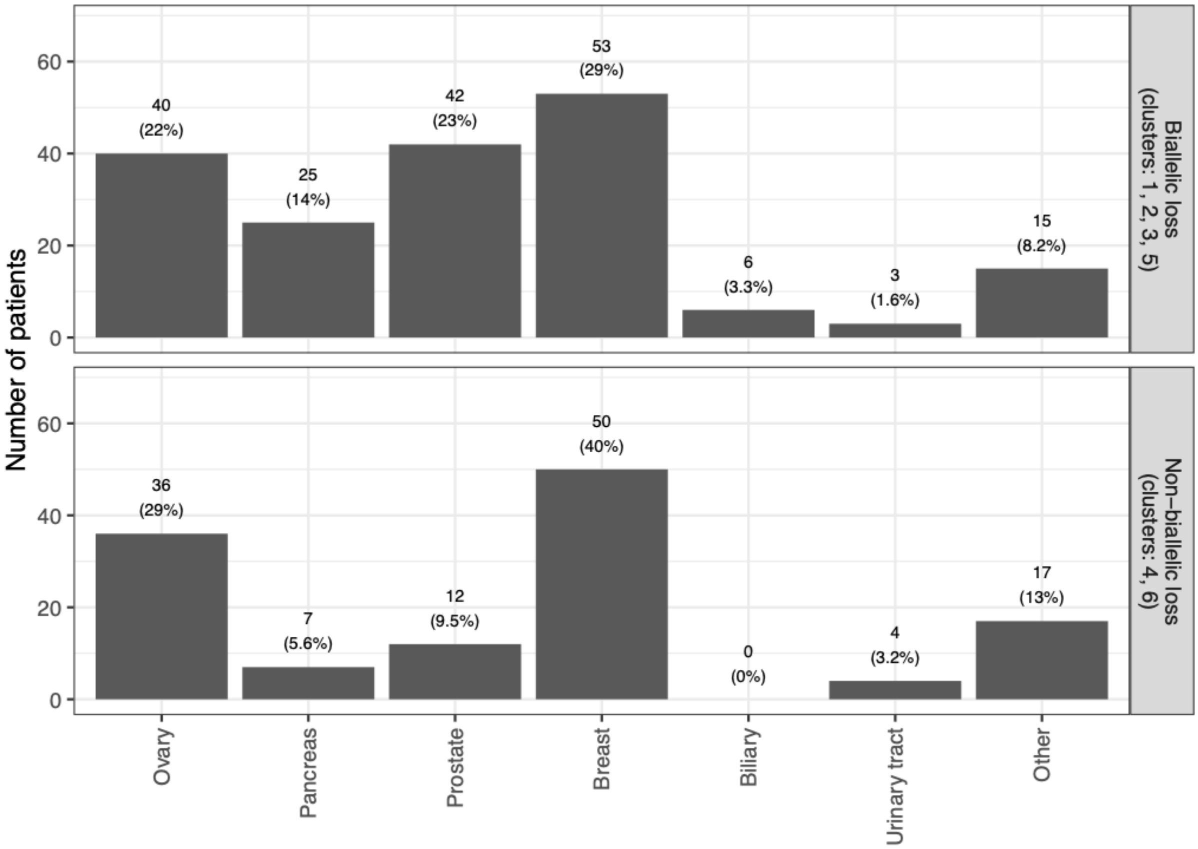

Supplementary figure 21: The number of CHORD-HRD patients of each cancer type which did (top) and did not (bottom) have biallelic loss of *BRCA1*, *BRCA2*, *RAD51C*, or *PALB2*. The top panel corresponds to patients in cluster 1,2,3 and 5 of **Figure 3c** while the bottom panel corresponds to patients in cluster 4 and 6. The percentages shown are within-group proportions.

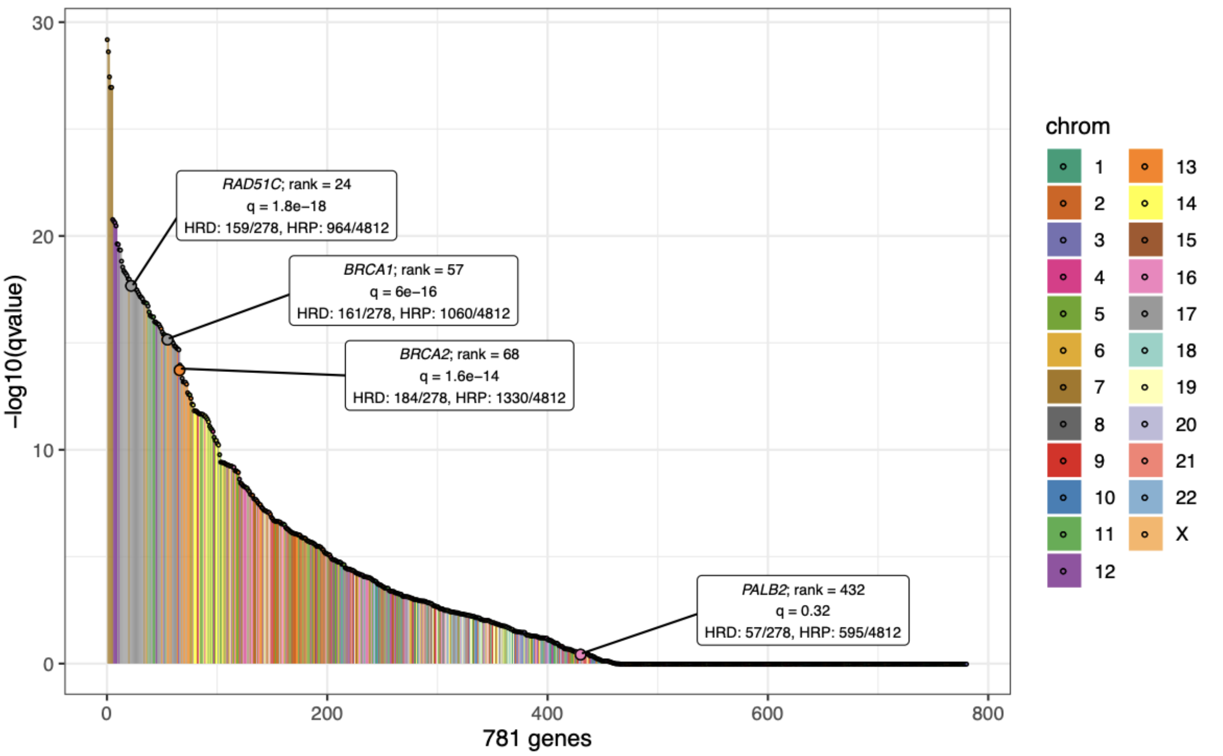

Supplementary figure 22: Enrichment of LOH in the 781 HR/cancer related genes. Enrichment for each gene was determined between CHORD-HRD samples (excluding those with deep deletions in *BRCA1/2*, *RAD51C* or *PALB2*) and CHORD-HRP samples using one-sided Fisher's exact tests.

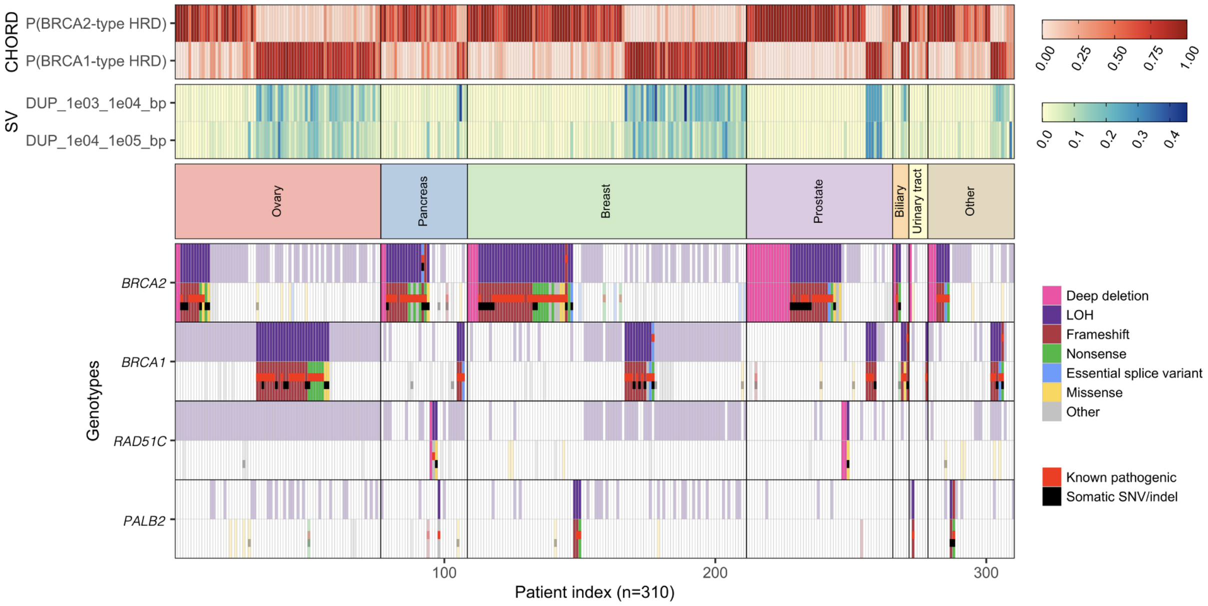

Supplementary figure 23: Biallelic status of *BRCA2*, *BRCA1*, *RAD51C* and *PALB2* in CHORD-HRD patients from both the HMF and PCAWG datasets. Patients were clustered both by cancer type and by HRD type. Top: *BRCA1*- and *BRCA2*-type HRD probabilities from CHORD. Middle: SV contexts used by CHORD to distinguish *BRCA1*- from *BRCA2*-type HRD. Bottom: The biallelic status of each gene. Tiles marked as ‘Known pathogenic’ refer to variants having a ‘pathogenic’ or ‘likely pathogenic’ annotation in ClinVar. Only data from samples that passed CHORD’s QC criteria are shown in this figure (MSI absent, ≥50 indels, and ≥30 SVs if a sample was predicted HRD). LOH: loss-of-heterozygosity

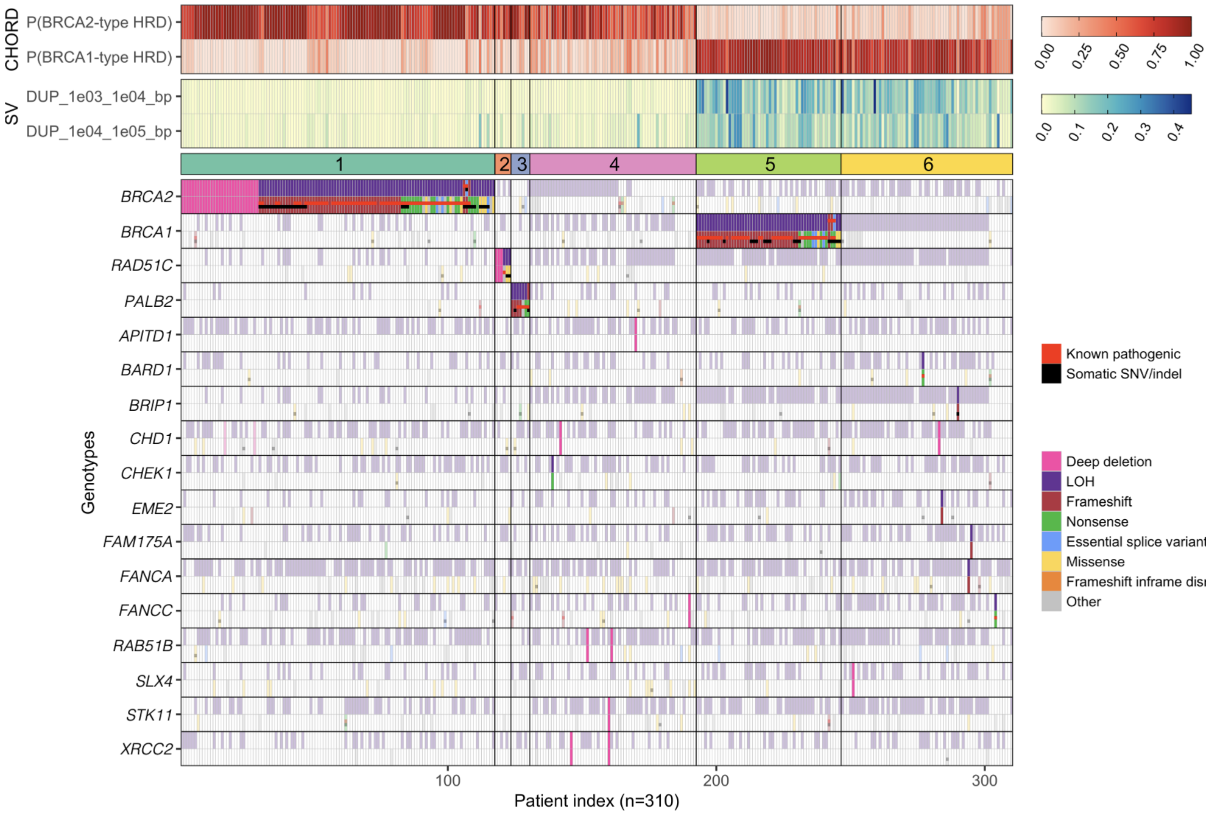

Supplementary figure 24: Biallelic status of *BRCA2*, *BRCA1*, *RAD51C*, *PALB2*, as well as other HR genes in CHORD-HRD patients from both the HMF and PCAWG datasets. Top: *BRCA1*- and *BRCA2*-type HRD probabilities from CHORD. Middle: SV contexts used by CHORD to distinguish *BRCA1*- from *BRCA2*-type HRD. Bottom: The biallelic status of each gene. Tiles marked as 'Known pathogenic' refer to variants having a 'pathogenic' or 'likely pathogenic' annotation in ClinVar. Only HR genes with one of the following events in at least one patient from cluster 4 or 6 was shown here: deep deletion; or LOH in combination with a pathogenic/likely pathogenic variant or a frameshift/nonsense variant. Only data from samples that passed CHORD's QC criteria are shown in this figure (MSI absent, ≥50 indels, and ≥30 SVs if a sample was predicted HRD). Furthermore, only genes where at least one patient had an impactful biallelic event are shown. LOH: loss-of-heterozygosity

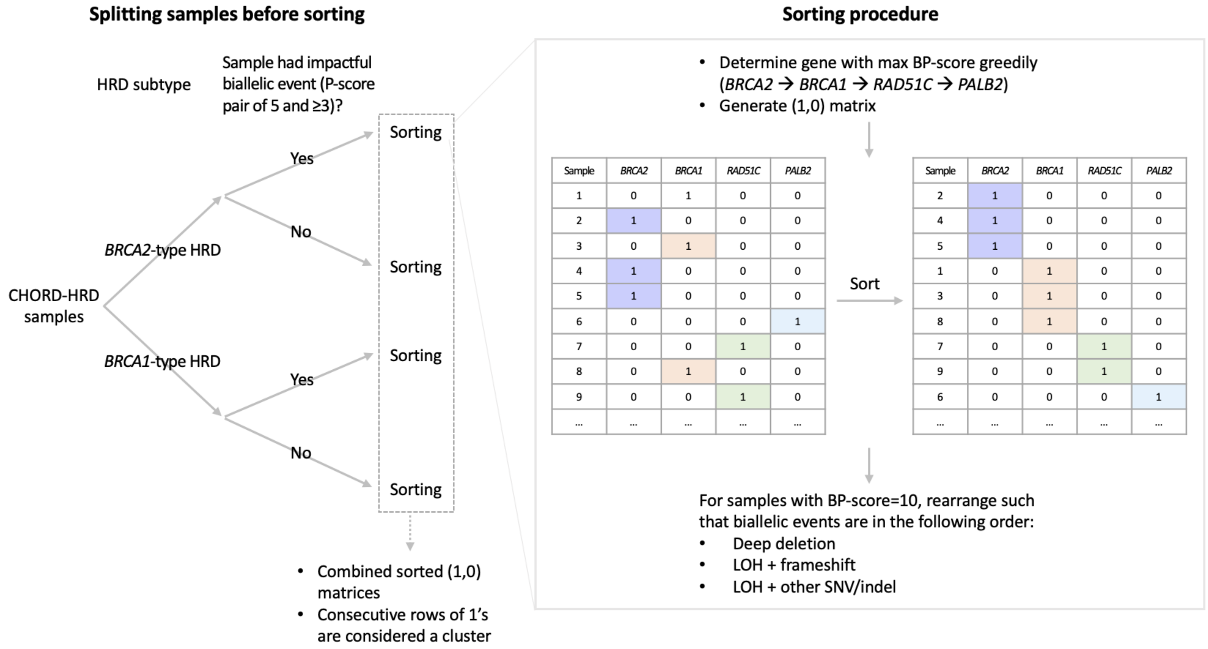

Supplementary figure 25: Overview of the procedure to sort and cluster CHORD-HRD samples. CHORD-HRD samples were first split by HRD subtype (*BRCA1*- or *BRCA2*-type HRD), and further split by whether a sample had an impactful biallelic event (defined as having a P-score pair of 5 and >=3). This produced 4 distinct groups of samples, which were sorted such that samples were ordered by deficiency of *BRCA2, BRCA1, RAD51C* and *PALB2* (in that order). This gene order corresponds to the enrichment significance of these genes as shown in **Figure 3b**. For each gene, samples were sorted such that samples with the HRD causing gene deficiency due to deep deletions were ordered first, followed by those with LOH (loss-of-heterozygosity) + frameshift and LOH + SNV/indel.

Supplementary notes

1. Prerequisites for accurate HRD prediction

It is important to note that microsatellite instability (MSI) negatively affects CHORD's ability to accurately predict HRD. MSI is a hypermutator phenotype characterized by an exceptional number of indels in regions with (tandem) repeats. As CHORD uses relative values of mutation contexts as input, MSI (defined as having more than 14,000 indels within repeat regions) results in a reduction of the relative contribution of microhomology deletions and thus an underestimated HRD probability ('false negatives'). **Supplementary figure 26** shows the CHORD being applied to all 3,824 HMF samples. All MSI samples were predicted HRP by CHORD even though 4 of these samples had biallelic loss of *BRCA2*. This could be circumvented by incorporating a MSI trained HRD classifier within CHORD. However, the number of HRD predicted samples with MSI is currently too small for training such a classifier and therefore we have built in MSI status checking as a quality control (QC) step within CHORD.

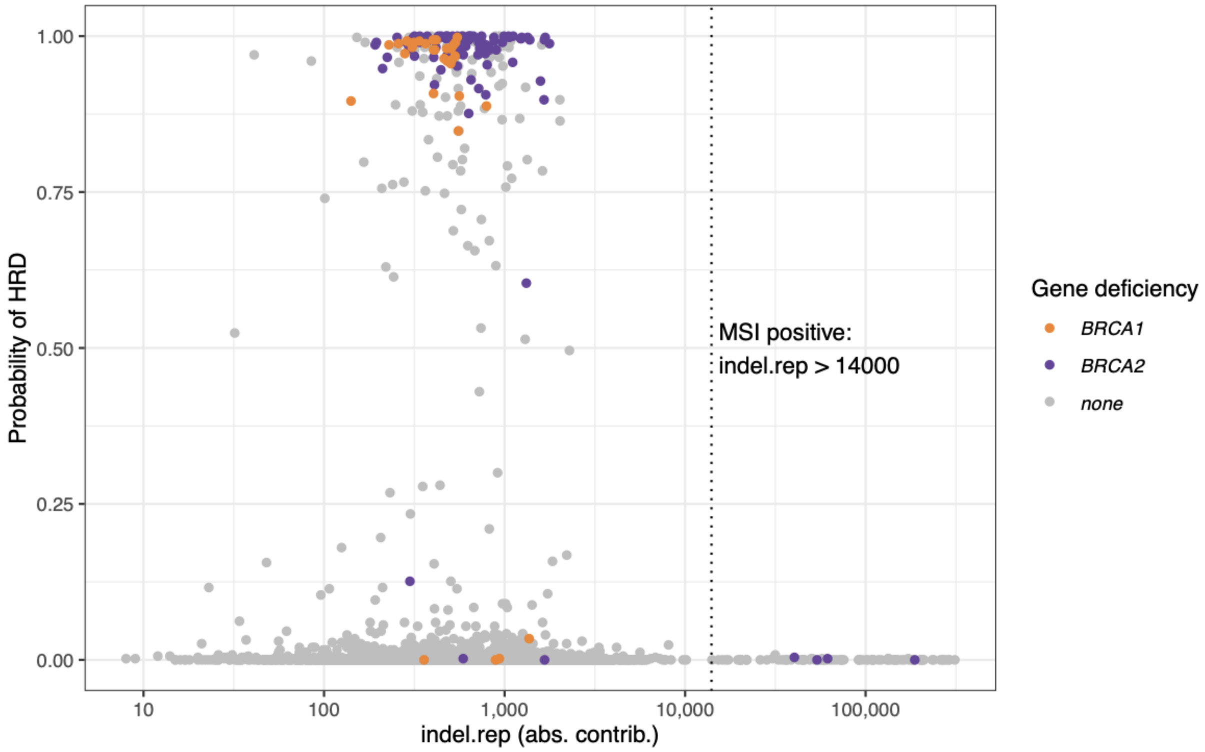

Supplementary figure 26: Microsatellite instability (MSI) impacts HRD prediction by CHORD. After applying CHORD to all 3824 tumors of the HMF dataset, all MSI positive tumors (those with more than 14,000 indels within repeat regions) had a low HRD probability, including 4 with *BRCA2* biallelic loss.

A minimum number of mutations is also required for accurate HRD prediction. To determine this, we progressively down-sampled all mutations for each sample in the training set and measured the reduction in performance (**Supplementary figure 27**). We found that at least 50 indels were required for accurately predicting HRD, and if a sample was predicted HRD, at least 30 SVs were required for distinguishing *BRCA1*-type from *BRCA2*-type HRD. These threshold levels may be particularly relevant for samples with low tumor purity and/or read coverage, and are as such also included as a QC step within CHORD.

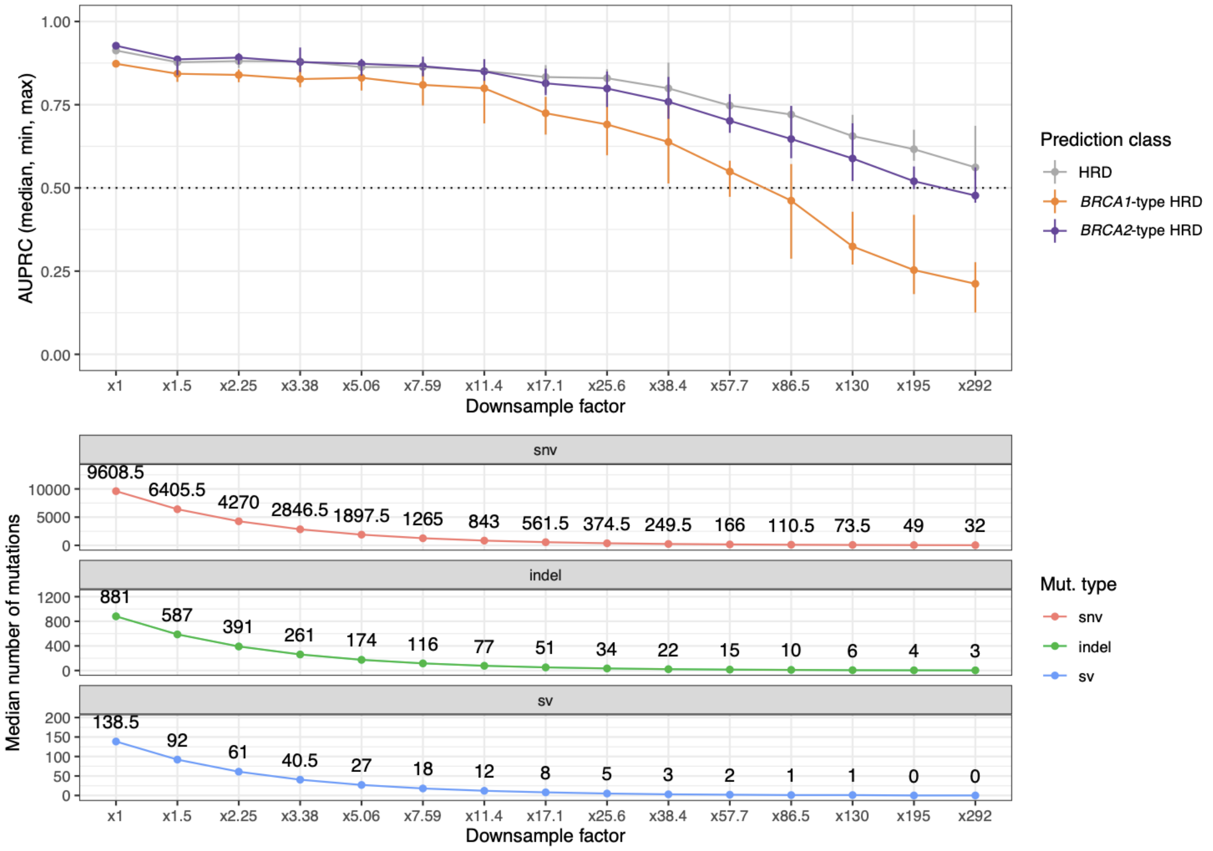

Supplementary figure 27: Performance of predicting HRD declines below ~50 indels. Similarly, performance for distinguishing *BRCA1*-type from *BRCA2*-type HRD declines below ~30 SVs. The set of mutations in each sample in the training set (used for training CHORD) were progressively downsampled. At each stage, CHORD was applied to the downsampled set of mutations, and thereafter, the area under the precision-recall curve was calculated.

2. CHORD as a tool to uncover novel pathogenic variants

The HRD predictions from CHORD can provide supportive evidence for interpreting variants of unknown significance (VUS), either germline or somatic, which can in turn be used for prioritizing rare VUS's for experimental validation as shown in **Supplementary figure 28**.

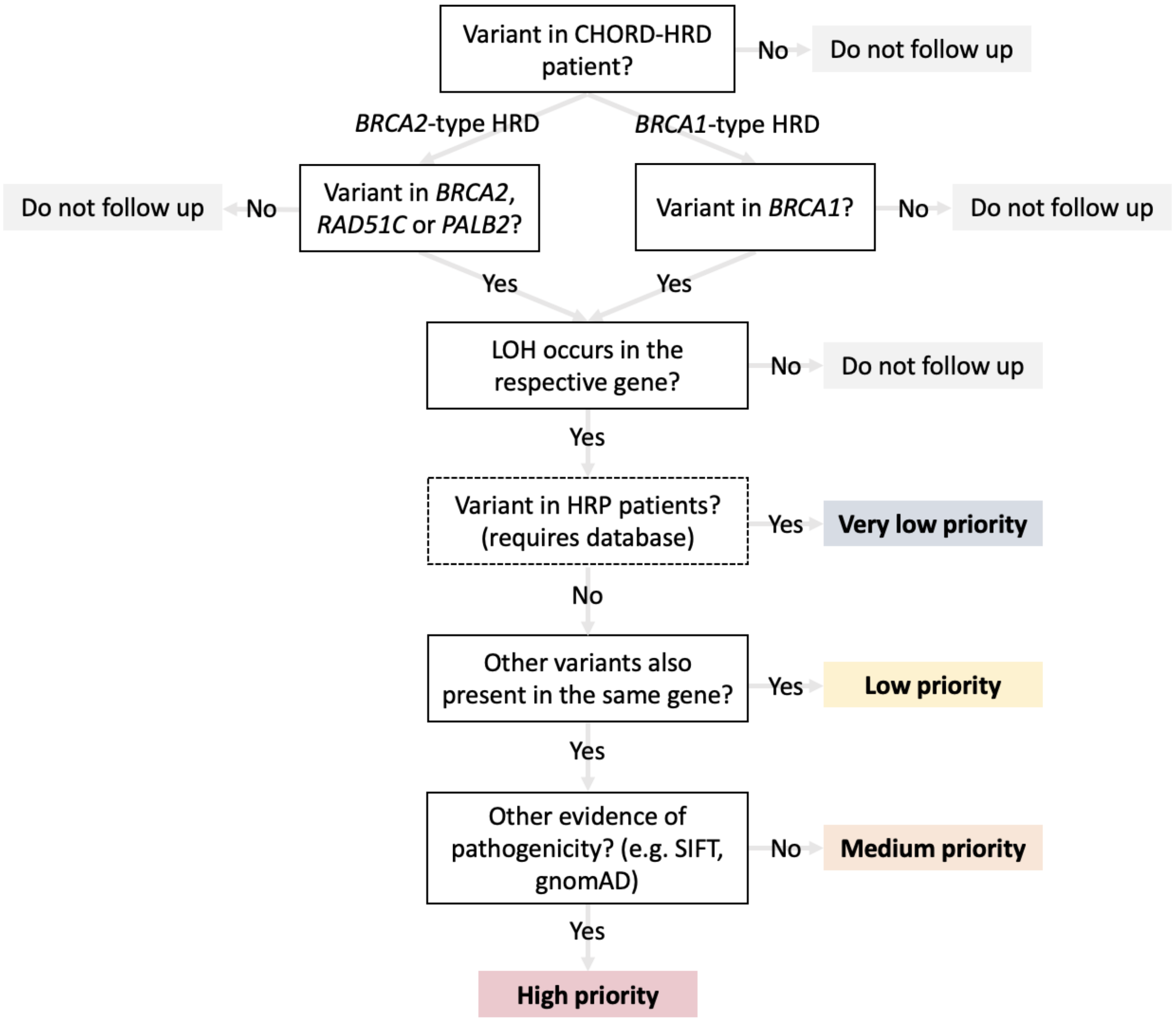

Supplementary figure 28: Scheme for prioritizing variants of unknown significance (VUS) for experimental validation starting from the HRD predictions from CHORD.

From 13 CHORD-HRD patients, we identified 14 variants not previously described to be pathogenic but which in combination with LOH could explain biallelic loss of a HR gene (**Supplementary data 7**). Moreover, biallelic loss of the respective gene corresponded to the associated HRD subtype, and all of these variants were not present in HRP samples, providing additional support that these variants are potentially pathogenic. From these variants, 2 *BRCA2* missense variants (c.9230T>C, c.9254C>T) were both found in patient HMF000429. Here, further validation is required to determine which was the driver mutation (or possibly the combination). The remaining 12 variants were the sole variant found in the respective gene and respective patient, and are thus more likely to be pathogenic driver variants. Of these, one *BRCA2* missense variant (c.8045C>T) had a 'Uncertain_significance' annotation in ClinVar but a low population frequency according to gnomAD as well as being predicted as 'deleterious' by SIFT and 'probably damaging' by PolyPhen, supporting its potential pathogenicity.

3. CHORD can detect HRD in a substantial number of cases that would be missed by genetic testing

To assess the potential value of CHORD in a clinical setting, we compared CHORD's predictions for HMF patients to the hypothetical outcomes of common genetic testing approaches. In the clinic, HRD detection is currently often done by screening for pathogenic *BRCA1/2* SNVs/indels based on annotations from curated databases (e.g. ClinVar). This is performed either on blood biopsies (blood genetic testing), analogous to screening for germline SNVs/indels in sequencing data of the HMF cohort (which was analysed by tumor-normal pair whole genome sequencing); or on tumor biopsies (tumor genetic testing), analogous to screening both germline and somatic SNVs/indels. Our genetic analyses (below figure) indicate that blood genetic testing would identify a pathogenic *BRCA1/2* SNV/indel (according to ClinVar; or an out-of-frame frameshift) in 18% of CHORD-HRD breast/ovarian cancer patients (which genetic testing is often restricted to), while tumor genetic testing would increase this proportion to 25%. If patients with other cancer types would be included, blood and tumor genetic testing would identify 31% and 42% of CHORD-HRD tumors (respectively) with a pathogenic *BRCA1/2* SNV/indel (**Supplementary figure 29a, columns 1 and 2**).

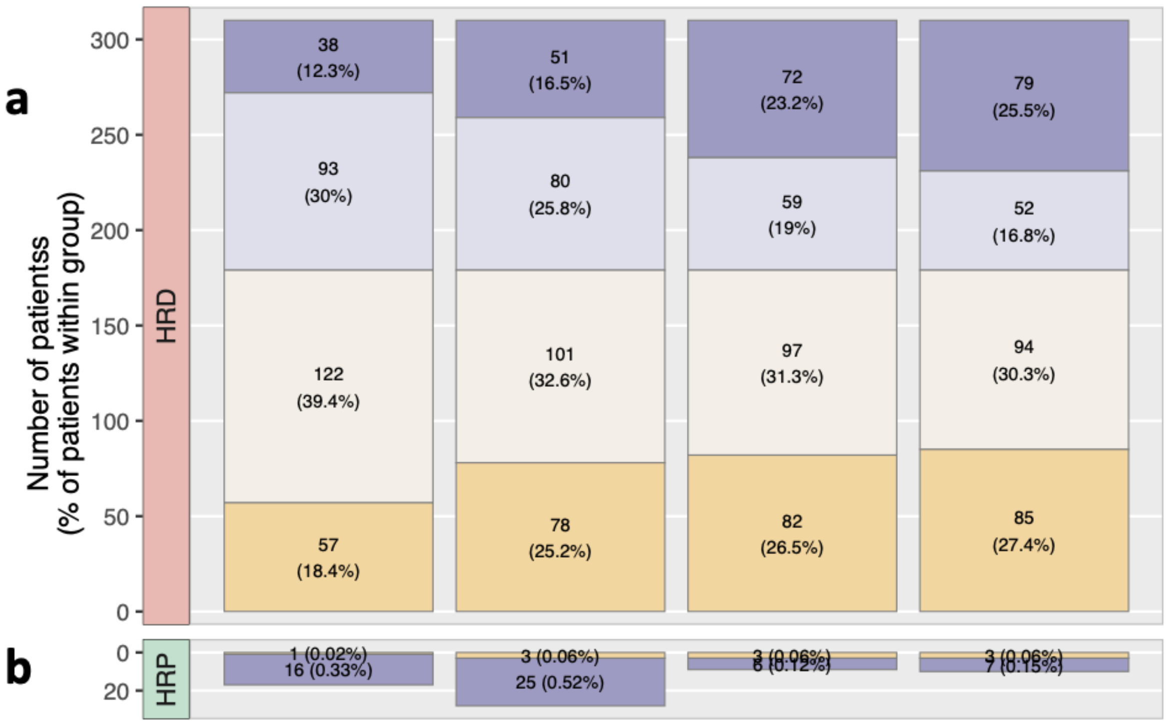

Genetic testing type	On blood	On tumor	WGS, matched blood/tumor	
Events tested for	*BRCA1/2* germline pathogenic mut.	*BRCA1/2* germline/somatic pathogenic mut.	*BRCA1/2* biallelic loss	*BRCA1/2, RAD51C, PALB2* biallelic loss

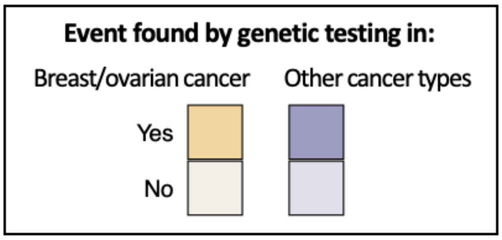

Supplementary figure 29: CHORD identifies a large proportion of HRD patients that would be missed by genetic testing. (**a**) and (**b**) show the percentage of CHORD-HRD or CHORD-HRP patients

(respectively) from the HMF dataset in which a pathogenic event was found based on four genetic testing setups. In the first two setups (‘On blood’, ‘On tumor’), a pathogenic event was identified if a pathogenic SNV/indel was found on one allele, which was defined as a frameshift, or a likely pathogenic or pathogenic variant according to ClinVar. In the ‘WGS based testing’ setups, the pathogenic SNV/indel must also have occurred in combination with loss of heterozygosity (LOH); or, a deep deletion was identified. Only data from samples that passed CHORD’s QC criteria are shown in this figure (MSI absent, ≥50 indels, and ≥30 SVs if a sample was predicted HRD).

While not currently routinely performed in the clinic, WGS based genetic testing with matched blood/tumor biopsies (WGS genetic testing) would allow the detection of any event (including structural events such as LOH or deep deletions) that contributes to HR gene inactivation, and enables the determination of biallelic gene status. Our analyses show that WGS genetic testing would increase the number of patients that are considered HRD compared to SNV/indel-based blood/tumor genetic testing, with biallelic loss of *BRCA1/2* being identified in 27% of CHORD-HRD breast/ovarian cancer patients and in 50% of patients pan-cancer (**Supplementary figure 29a, column 3**). Additionally, WGS genetic testing would consider 9 CHORD-HRP patients as HRD (**Supplementary figure 29b, column 3**; ‘genetic testing false positives’; these could also be CHORD false negatives), a marked decrease compared to tumor genetic testing which would identify 28 false positives (**Supplementary figure 29b, column 2**). By including the two other main HRD associated genes (*RAD51C* and *PALB2*) in WGS genetic testing, biallelic gene loss would be identified in 53% of patients (**Supplementary figure 29a, column 4**), while only increasing the number of genetic testing false positives from 9 to 10 patients (**Supplementary figure 29b, column 5**). Our findings show that while WGS genetic testing (for biallelic loss) offers improved detection of HRD patients compared to testing for pathogenic SNVs/indels, it still misses around half of HRD patients as classified by CHORD.
