# Supplementary material for: Pan-cancer landscape of homologous recombination deficiency
Source: Nat Commun. 2020 Nov 4;11:5584. doi: 10.1038/s41467-020-19406-4 (PMC7643118; doi:10.1038/s41467-020-19406-4)
Supplement: Supplementary file 3 — Description of Additional Supplementary Files [file 41467_2020_19406_MOESM3_ESM.pdf]

Description of Additional Supplementary Files

File Name: Supplementary Data 1
Description: Predictions from CHORD and CHORD-signature on the HMF, BRCA-EU, and PCAWG datasets as well as metadata for each sample

File Name: Supplementary Data 2
Description: Mutation contexts and mutational signatures extracted from the HMF, BRCA-EU, and PCAWG datasets

File Name: Supplementary Data 3
Description: List of 781 cancer and HR related genes used for the pan-cancer analysis of HRD and results of the enrichment analysis to determine HRD associated genes. The enrichment analysis was performed using one-sided Fisher's exact tests with multiple testing correction using the Hochberg procedure

File Name: Supplementary Data 4
Description: Genotypes of *BRCA1/2, RAD51C, PALB2* and other HR genes for the 310 CHORD-HRD patients from the HMF and PCAWG datasets

File Name: Supplementary Data 5
Description: Incidence and genetic cause of HRD by cancer type in the HMF and PCAWG dataset

File Name: Supplementary Data 6
Description: Pathogenicity scoring of variants used to determine biallelic gene status, including biallelic pathogenicity score inclusion criteria for CHORD training data

File Name: Supplementary Data 7
Description: Details for the novel, potentially pathogenic variants of unknown significance (VUS)
