# Supplementary material for: Pan-cancer landscape of homologous recombination deficiency
Source: Nat Commun. 2020 Nov 4;11:5584. doi: 10.1038/s41467-020-19406-4 (PMC7643118; doi:10.1038/s41467-020-19406-4)
Supplement: Supplementary file 11 — Reporting Summary [file 41467_2020_19406_MOESM11_ESM.pdf]

nature research

Corresponding author(s): Edwin Cuppen

Last updated by author(s): Sep 18, 2020

Reporting Summary

Nature Research wishes to improve the reproducibility of the work that we publish. This form provides structure for consistency and transparency in reporting. For further information on Nature Research policies, see Authors & Referees and the Editorial Policy Checklist .

Statistics

For all statistical analyses, confirm that the following items are present in the figure legend, table legend, main text, or Methods section.

n/a	Confirmed	
☐	☒	The exact sample size (*n*) for each experimental group/condition, given as a discrete number and unit of measurement
☐	☒	A statement on whether measurements were taken from distinct samples or whether the same sample was measured repeatedly
☐	☒	The statistical test(s) used AND whether they are one- or two-sided<br>*Only common tests should be described solely by name; describe more complex techniques in the Methods section.*
☒	☐	A description of all covariates tested
☐	☒	A description of any assumptions or corrections, such as tests of normality and adjustment for multiple comparisons
☐	☒	A full description of the statistical parameters including central tendency (e.g. means) or other basic estimates (e.g. regression coefficient) AND variation (e.g. standard deviation) or associated estimates of uncertainty (e.g. confidence intervals)
☒	☐	For null hypothesis testing, the test statistic (e.g. *F*, *t*, *r*) with confidence intervals, effect sizes, degrees of freedom and *P* value noted<br>*Give P values as exact values whenever suitable.*
☒	☐	For Bayesian analysis, information on the choice of priors and Markov chain Monte Carlo settings
☒	☐	For hierarchical and complex designs, identification of the appropriate level for tests and full reporting of outcomes
☒	☐	Estimates of effect sizes (e.g. Cohen's *d*, Pearson's *r*), indicating how they were calculated

Our web collection on statistics for biologists contains articles on many of the points above.

Software and code

Policy information about availability of computer code

Data collection

WGS data and corresponding metadata have been obtained from external publicly available sources :

Metastatic cancer data was obtained from Hartwig Medical Foundation (HMF) and provided under data request number DR-47 and DR-10. Both WGS and clinical data is freely available for academic use from HMF through standardized procedures and request forms can be found at https://www.hartwigmedicalfoundation.nl. This data was consented for by patients as part of two clinical studies (NCT01855477, NCT02925234)

Somatic variant TSV files of the 560 breast cancer (BRCA-EU) dataset were downloaded from the International Cancer Genome Consortium (ICGC; https://dcc.icgc.org/repositories) in August 2017. BAM files for the 44 BRCA-EU samples are available from EGA (datasets: EGAD00001000063 [https://www.ebi.ac.uk/ega/datasets/EGAD00001000063], EGAD00001001322 [https://www.ebi.ac.uk/ega/datasets/EGAD00001001322], EGAD00001001337 [https://www.ebi.ac.uk/ega/datasets/EGAD00001001337]. BRCA1/2 status annotations for this dataset being obtained from the supplementary data in Davies et al 2017

Somatic variant VCF files and somatic copy-number TSV files for the ICGC portion of the Pan-Cancer Analysis of Whole Genomes (PCAWG) dataset were downloaded from https://dcc.icgc.org/releases/PCAWG on March 3, 2020. PCAWG access for germline data has been granted via the Data Access Compliance Office (DACO) Application Number DACO-1050905 on October 6, 2017 and via https://console.cancercollaboratory.org download portal on December 4, 2017. Germline VCF files were downloaded from the cancer collaboratory download portal on March 21, 2018.

Data analysis

Variant calling was performed using the in-house pipeline at HMF: https://github.com/hartwigmedical/pipeline, https://github.com/hartwigmedical/hmftools. This pipeline uses several external packages:
- BWA-MEM v0.7.5a: read mapping
- GATK v3.4.46 IndelRealigner: indel realignment
- GATK v3.4.46 Haplotype Caller: calling germline variants in the reference sample.

- GATK v3.4.46 BQSR3: recalibrate base qualities
- Strelka v1.0.14: somatic SNV and indel variant calling.
- GRIDSS v1.8.0: somatic SV calling
- PURity & PLoidy Estimator (PURPLE): copy-number calling

All code used for training CHORD and for data analysis used is publicly available and can be found at https://github.com/UMCUGenetics/CHORD/tree/2.00.
Supporting code for CHORD can be found at the following github repos:
https://github.com/UMCUGenetics/mutSigExtractor/tree/1.14 (extraction of mutational patterns)
https://github.com/UMCUGenetics/mltoolkit (classification statistics)
https://github.com/UMCUGenetics/hmfGeneAnnotation/tree/2.02 (determining biallelic gene status)

For manuscripts utilizing custom algorithms or software that are central to the research but not yet described in published literature, software must be made available to editors/reviewers. We strongly encourage code deposition in a community repository (e.g. GitHub). See the Nature Research guidelines for submitting code & software for further information.

Data

Policy information about availability of data

All manuscripts must include a data availability statement. This statement should provide the following information, where applicable:
- Accession codes, unique identifiers, or web links for publicly available datasets
- A list of figures that have associated raw data
- A description of any restrictions on data availability

See above at "Software and Code - Data Collection"

Field-specific reporting

Please select the one below that is the best fit for your research. If you are not sure, read the appropriate sections before making your selection.

- [x] Life sciences
- [ ] Behavioural & social sciences
- [ ] Ecological, evolutionary & environmental sciences

For a reference copy of the document with all sections, see nature.com/documents/nr-reporting-summary-flat.pdf

Life sciences study design

All studies must disclose on these points even when the disclosure is negative.

Sample size	The metastatic tumor sample cohort (HMF cohort) described in the paper consists of 3824 independent samples from 3584 patients (all patients in the cohort).<br>The primary breast cancer cohort (BRCA-EU, ICGC) which was used for independent performance validation CHORD consisted of unique tumors from 371 patients. These patients were those where the BRCA1/2 biallelic status could confidently be determined as per the gene annotations from Davies et al 2017<br>The primary pan-cancer tumor cohort (PCAWG cohort) consisted of unique tumors from 1854 patients. These patients were part of the ICGC portion of the PCAWG dataset and for which we could obtain access to the germline variants
Data exclusions	320 HMF were excluded from the pan-cancer analysis of HRD that were duplicate tumors (for patients with multiple biopsies), or failed CHORD's QC criteria. Accurate HRD prediction with CHORD requires samples with >=50 indels, >=30 SVs and not MSI.<br><br>From a total of 2583 PCAWG samples with data of optimal quality, 729 samples (TCGA portion) were excluded as we did not have access to the germline data for these samples. An additional 236 were excluded due to failing CHORD QC as indicated above.
Replication	Independent cancer cohorts were used for performance validation of CHORD.
Randomization	Not relevant for this study.
Blinding	Not relevant for this study.

Reporting for specific materials, systems and methods

We require information from authors about some types of materials, experimental systems and methods used in many studies. Here, indicate whether each material, system or method listed is relevant to your study. If you are not sure if a list item applies to your research, read the appropriate section before selecting a response.

Materials & experimental systems

n/a	Involved in the study
☒	☐ Antibodies
☒	☐ Eukaryotic cell lines
☒	☐ Palaeontology
☒	☐ Animals and other organisms
☒	☐ Human research participants
☒	☐ Clinical data

Methods

n/a	Involved in the study
☒	☐ ChIP-seq
☒	☐ Flow cytometry
☒	☐ MRI-based neuroimaging
